# Priority effects during fungal community establishment in beech wood

**DOI:** 10.1038/ismej.2015.38

**Published:** 2015-03-20

**Authors:** Jennifer Hiscox, Melanie Savoury, Carsten T Müller, Björn D Lindahl, Hilary J Rogers, Lynne Boddy

**Affiliations:** 1 School of Biosciences, Cardiff University, Sir Martin Evans Building, Cardiff CF10 3AX, UK; 2 Department of Soil and Environment, Swedish University of Agricultural Sciences, Uppsala, Sweden

## Abstract

Assembly history of fungal communities has a crucial role in the decomposition of woody resources, and hence nutrient cycling and ecosystem function. However, it has not been clearly determined whether the fungal species that arrive first may, potentially, dictate the subsequent pathway of community development, that is, whether there is a priority effect at the species level. We used traditional culture-based techniques coupled with sequencing of amplified genetic markers to profile the fungal communities in beech (*Fagus sylvatica*) disks that had been pre-colonised separately with nine species from various stages of fungal succession. Clear differences in community composition were evident following pre-colonisation by different species with three distinct successor communities identified, indicating that individual species may have pivotal effects in driving assembly history. Priority effects may be linked to biochemical alteration of the resource and combative ability of the predecessor.

## Introduction

Community structure is a key driver of ecosystem dynamics (Deacon *et al.*, 2006; Hansen *et al.*, 2008). However, variation in ecophysiological properties of decomposer communities often confound models predicting carbon cycling and other ecosystem functions (Bardgett *et al.*, 2008; Chapin *et al.*, 2009), because changes in decomposer identities are often idiosyncratic and difficult to predict, as well as being highly sensitive to environmental variation (Wardle, 2002; Heichmann and Reichstein, 2008). In ecosystem models, the microbial community is often considered a 'black box' (Andren and Balandreau, 1999), and community structure is omitted despite the fact that understanding decomposer community dynamics is critical for elucidating the processes underlying carbon dynamics (McGuire and Treseder, 2010). Wood-decay fungi are key determinants of decomposition of recalcitrant lignocellulose and, therefore, of nutrient cycling and carbon sequestration rates in forest ecosystems (Baldrian and Lindahl, 2011). Neglect of fungal community composition and dynamics may lead to major discrepancies between observed and predicted decay rates in models of wood decomposition (Radtke *et al.*, 2009; Zell *et al.*, 2009; Palviainen *et al.*, 2010; Woodall, 2010; Van der Wal *et al.*, 2014).

Assembly history (the timing and sequence in which species join a community) has a large influence on community structure and function in decomposer communities (Fukami *et al.*, 2010; Dickie *et al.*, 2012; Ottosson *et al.*, 2014). Simply put, the identity and abundance of species that first colonise an environment may affect the colonisation success of species that arrive later, and thus determine the structure of the community. Such 'priority effects' likely have a major role in explaining the variation in the structure of communities found in different habitats with similar environmental conditions (Chase, 2010; Weslien *et al.*, 2011). Wood-decay fungi are ideal for studies of assembly history and priority effects; it is well established that some species colonise wood earlier than others, but there is large stochastic variation in the timing of species immigration and the interactions between species within woody resources (Boddy, 2001; Boddy and Heilmann-Clausen 2008; Fukami *et al.,* 2010).

Early colonisers of wood are often ruderal opportunists arriving as spores, or endophytes latently present in functional sapwood, which develop overtly forming communities fairly characteristic for different angiosperm tree species (Boddy *et al.,* 1989; Hendry *et al.,* 2002; Parfitt *et al.,* 2010). Later colonisers arrive as spores or via the soil as mycelium, often aggregated to form cords or rhizomorphs (Fricker *et al.,* 2008; Boddy *et al.,* 2009). Fungal community change most commonly results from antagonistic interactions, but also from changes in the microclimatic environment (Boddy and Heilmann-Clausen, 2008). Mycelial antagonism results either in deadlock (where there is no change in territory occupied by either combatant) or replacement (partial or complete) of one combatant by another, leading to community change (Boddy, 2000). The intial community will gradually alter as species are displaced by more aggressive 'secondary' colonisers, which may in turn be replaced by even more combative species and by stress-tolerant species (Holmer and Stenlid, 1997; Boddy, 2001; Boddy and Heilmann-Clausen, 2008).

Different species vary in the rate and ways in which they decompose wood, for example, in the relative proportion and location of substrates used, alteration of physical properties or the production of secondary metabolites (Worrall *et al.,* 1997; Boddy, 2000; Boddy and Heilmann-Clausen, 2008; Woodward and Boddy, 2008). Decaying wood can, thus, be thought of as a three-dimensional mosaic of interspecific interactions and abiotic conditions manipulated by the fungi within. Alteration of the resource will affect both current and subsequent inhabitants. Different predecessor species may, therefore, effectively select for successor species that are adapted to certain conditions. For example, circumstantial evidence for priority effects are provided by co-occurring pairs of predecessors/successors, identified in fruit body surveys (Ottosson *et al.*, 2014).

To assess priority effects in wood-decay communities accurately, the abundance and diversity of species following on from different individual preceding species must be determined experimentally. To date, studies of priority effects have either used few initial species (for example, Lindner *et al.,* 2011), or examined the effects of several pre-colonisers on a fixed set of successor species (for example, Fukami *et al.,* 2010; Dickie *et al.,* 2012). Here we test the hypothesis that priority effects determine fungal community composition in wood, by pre-colonising beech disks with one of nine species from different successional stages and placing them on the floor of a deciduous woodland for up to 24 months, followed by characterisation of the resulting communities using culture- and incubation-based approaches coupled with high-throughput sequencing of amplified ITS2 markers. We also test the hypotheses that community development is affected by time in the field, season of exposure and the decay state of the resource.

## Materials and methods

### Colonisation of wood disks

Cultures of nine native, beech (*Fagus sylvatica*)-inhabiting fungi (Table 1), representing species from the primary, secondary and late secondary/tertiary stages of community succession (Boddy and Heilmann-Clausen, 2008 and references within), were maintained on 0.5% MA (malt agar: 5 g l^−1^ malt extract, 15 g l^−1^ agar no. 2; LabM, Heywood, UK) at 20 °C in the dark. Beech wood disks (diameter 10 cm, thickness 2 cm) were cut from freshly felled branches and sterilised by autoclaving three times at 126 °C over a 72-h period. Sterile disks were colonised by placing onto mycelia growing on 0.5% MA in plastic 400-ml deli pots (Cater4you, High Wycombe, UK). Holes (4 × 0.8 mm^2^) covered in microporous tape provided aeration. Pots were incubated at 20 °C in the dark for 12 or 24 weeks. Initial density of pre-colonised disks was determined as oven-dry-weight per fresh volume, and pH was measured after shaking 0.5 g sawdust in 5 ml distilled water for 1 h.

### Field site characteristics and experimental layout

The site was a mixed deciduous woodland dominated by *F. sylvatica* in Wytham Great Wood (Oxford University; 51.77727, −1.341255). A 25 × 25-m grid, divided into 10 × 10 sections, was marked on the site and experimental units allocated to different squares. Uncolonised, sterile disks and colonised disks, scraped free of adhering mycelium, were placed in the litter layer, distributed across the site grid in a randomised block design, such that each pre-coloniser treatment occurred only once in each row/column (Supplementary Figure 1). Each pre-coloniser species/treatment had 10 replicates; multiple disks from different subexperiments were placed at each sample location.

The effect of length of time in the field on fungal community development was assessed by harvesting disks, which had been placed out in the field in September 2011, every 6 months over 24 months (experiment A1). Further disks were placed in the field in December 2011, March 2012 and June 2012, and harvested after 6 and 12 months to assess the effect of season of release (experiment A2). To assess the effect of length of pre-colonisation, disks that had been pre-colonised for 12 or 24 weeks were placed in the field in September 2012 and harvested after 12 months (experiment B). The effect of short-term variation in release date was assessed by placing disks in the field at 2-week intervals over 8 weeks beginning September 2011 and harvesting after 6 months (experiment C). All experiments are detailed in Table 2.

### Isolation, DNA sample generation and direct incubation

After harvest, adhering litter/soil were removed from disks, characteristics such as zone lines and size were noted and any attached mycelial cords sampled by placing small sections onto 2% MA following surface sterilisation (10% sodium hypochlorite for 30 s). Both sides of the disks were photographed using a Coolpix P560 camera (Nikon UK Ltd, Surrey, UK). Disks were surface-sterilised by dipping in 10% sodium hypochlorite for 30 s, and six 1–2-mm chips were removed from each face using a 6-mm sterile chisel; these chips were placed aseptically onto 2% MA and incubated at 20 °C in the dark until mycelia had emerged. Where present, pre-coloniser fungi were identified based on colony mycelial morphology on agar (which were all distinctively different based on colour, extension rate, character of aerial mycelium and so on) and any unknown mycelia were subcultured onto 2% MA. Subsequently, disks were drilled through their whole width at 20+ points using a sterile 4-mm drill bit and the resulting sawdust was stored at −20 °C until use. Disks were then sprayed with distilled water and incubated at 20 °C in the dark for 1–2 months to allow outgrowth of mycelium.

### Molecular identification of unknown fungi

DNA was extracted from unknown mycelia isolated into pure culture, and from outgrowing mycelia and cords attached to wood disks, using the method described by Cenis (1992) modified to include 0.4% w/v skimmed milk in the initial extraction buffer. PCR amplifications were performed using the ITS1F/ITS4 (Gardes and Bruns, 1993) primer combination following Parfitt *et al.* (2010). PCR products were purified using Qiagen PCR purification kits (Qiagen, Manchester, UK) and sequenced using the 3710 × l DNA analyser with Big Dye Terminator v3.1 (Life Technologies Ltd, Paisely, UK) by Eurofins Genomics (Ebersberg, Germany). Sequences were identified by comparison with all fungal sequences in the UNITE and INSD databases by BLASTn using the massBLASTer function in PlutoF (Abarenkov *et al.*, 2010). Where fungi could not be identified through ITS sequencing, they were grouped by similar mycelial morphology into 'cultured operational taxonomic units' (cOTUs) and assigned an identification number.

### Preparation of samples for 454 pyrosequencing

454 sequencing of amplicons was performed on 72 samples: seven pre-coloniser species plus uncolonised controls (nine replicates), from disks placed out in September 2011 and collected September 2012 (experiment A). DNA was extracted from sawdust samples using the PowerSoil DNA extraction kit (MoBio, Carlsbad, CA, USA) with the addition of an initial bead-beating step to aid tissue lysis (3 × (4 ms^−1^ for 20 s); FastPrep-24, MP Biomedicals, Santa Ana, CA, USA). PCR amplifications of the ITS2 region were conducted using the ITS4 primer extended with 8-bp sample identification tags (designed by Ihrmark *et al.,* 2012; high-performance liquid chromatography-purified; Integrated DNA Technologies Inc., Leuven, Belgium; Supplementary Table 1) in combination with the primer gITS7 (Ihrmark *et al.,* 2012). PCR was performed using a Veriti thermal cycler (Life Technologies Ltd) in 50-μl reactions (0.25 ng template, 200 μM of each nucleotide, 300 nM tagged-ITS4, 500 nM gITS7, 0.025 U μl^−1^ Taq polymerase (DreamTaq, Thermo Scientific, Waltham, MA, USA) in buffer; 5 min at 94 °C; 22–30 × (30 s at 94 °C; 30 s at 56 °C; 30 s at 72 °C); 7 min at 72 °C). Cycle numbers were optimised to ensure reactions were stopped in the early stages of the log phase, as gITS7 contains degenerate bases in two positions, potentially leading to biased amplicon composition at high cycle numbers.

Triplicate PCRs were performed for each sample, combined and electrophoresis performed in ultrapure agarose (Life Technologies Ltd) prior to excision and purification of bands using the Qiaquick gel extraction kit (Qiagen). Quantification was performed using the Quant-iT PicoGreen dsDNA assay kit (Life Technologies Ltd), following which equal amounts of PCR product from each sample were merged into two amplicon libraries. Each amplicon library was sequenced on ¼ plate using the Roche GS FLX+ 454 pyrosequencing platform (Hoffman La-Roche Ltd, Basel, Switzerland) by the NERC-Biomolecular Analysis Facility (Centre for Genomics Research, Liverpool, UK).

### Sequence analysis

Sequences were analysed using the SCATA pipeline (scata.mykopat.slu.se; Ihrmark *et al.*, 2012). Sequences were filtered by screening for primer sites and removing any sequences with an average quality score below 20, or with a score below 10 at any position. This resulted in 257 189 high-quality sequences, which were then compared for similarity using BLAST as a search engine, with a pairwise alignment scoring function with 1 in penalty for mismatch, 0 for gap opening and 1 for gap extension. Homopolymers were collapsed to 3 bp before clustering. Sequences were assembled into clusters by single-linkage clustering with a minimum of 99% similarity to the nearest neighbour demanded for sequences to enter clusters. Sequences that only occurred once in the entire data set (global singletons) were excluded in further analyses, as were clusters with fewer than two occurrences (<1% total sequences per sample). Representative sequences of all clusters (operational taxonomic units (OTUs)) were compared with all fungal sequences in the UNITE and INSD databases by BLASTn using the massBLASTer function in PlutoF (Abarenkov *et al.,* 2010). Taxonomic information for each OTU was obtained using the Galaxy project toolkit (http://usegalaxy.org/). Sequence data are archived at NCBI SRA under accession no. SRP052547.

### Statistical analysis

All statistical analyses were performed using R v3.1.0 (R Core Team, 2013), using the vegan package (Oksanen *et al.,* 2013), unless otherwise stated, and graphs generated using the package ggplot2 (Wickham, 2009). Fungal diversity estimates (Shannon diversity, Fisher's alpha and Pielou's evenness) were compared across pre-coloniser treatments using one-way analysis of variance (ANOVA). Differences in fungal community composition between treatments were visualised using non-metric multidimensional scaling. Samples with less than 205 sequences were excluded from the analysis, and the remaining samples rarefied to the lowest number of sequences in any sample (205). OTUs corresponding to pre-coloniser species were removed from the data set before ordination (removal of pre-colonisers prior to rarefying did not alter significance of results; Supplementary Table 2). The required distance matrices were constructed using the Bray–Curtis dissimilarity index (Clarke and Warwick, 2001). Analysis was conducted for rarefied raw data (assessment of random changes in most abundant taxa) following fourth-root transformation (to reduce the influence of the most abundant taxa relative to less dominant taxa and allow community-wide assessment of changes in taxon composition; Clarke and Warwick, 2001) and using non-rarefied data (to ensure rarefying did not alter the overall outcome; Supplementary Table 2). As there were no differences in overall outcomes, the untransformed rarefied data were used for subsequent analyses.

Permutational multivariate ANOVA (adonis function, 999 permutations) was used to assess whether treatment groupings apparent in non-metric multidimensional scaling plots were significantly different, with betadisper tests used to confirm equal dispersion between treatment groups. Pairwise tests were then used to compare differences between individual groups, and *P*-adjustment performed (Benjamini and Hochberg (1995) method). Taxa responsible for driving changes in community composition between groups were identified using similarity percentage analysis. OTUs were divided into those identified as basidiomycete species actively contributing to wood decomposition (decomposers), and ascomycetes supposedly unable to effect white or brown rot, instead living opportunistically off other mycelia (co-colonisers; Supplementary Table 3). The above analyses were run on both groups separately to detect whether there were any changes in the community structure of decomposer vs co-coloniser species following different pre-colonisers. Mantel tests (vegan) were performed to detect whether community composition was linked to position on the site or disk area/volume (correlation between a Euclidean distance matrix and the Bray–Curtis dissimilarity matrix; Legendre and Legendre, 1998). For fungal isolates, the frequency of occurrence of invading (that is, non-pre-coloniser) fungi (cOTUs) between different pre-coloniser species were assessed by pooling data from each subexperiment by species (each subexperiment acting as a replicate), then using permutational multivariate ANOVA and betadisper tests as described previously, with differences in cOTU communities between pre-coloniser species visualised by classical multidimensional scaling using the function cmdscale.

## Results

### Isolation of fungi from wood disks

The retrieval rate of disks across all experiments was 90.6% (Supplementary Table 4). Fungal and/or bacterial outgrowth occurred from all isolation points, with 38.1% chips resulting in outgrowth of two or more species. The original coloniser was still present in 42.8% of the disks (29.6% of isolation points); invading fungi were isolated from 92.8% of the disks (66.2% of isolation points) indicating at least partial replacement of, or co-colonisation with, the pre-coloniser. Of the unknown 'invading' fungi detected, most (94% of isolation points) were considered to be opportunistic species, not active in wood decay, which grew and sporulated very quickly on agar and may have colonised the surface of the disk only. Those that occurred most frequently were identified by sequencing as *Hypocrea avellanea*, *Mucor* sp. and *Rhizopus* sp. Invading species rarely captured a whole disk; only 0.8% of all disks exhibited outgrowth of the invader from every isolation point.

On the basis of reisolation, retention of all pre-colonisers, except *Stereum hirsutum*, decreased with increasing time in the field (experiment A1; Table 2), and the early-successional species *V. comedens, H. fragiforme* and *B. nummularia* were not detected after 12 months. There was a reciprocal relationship between the isolation frequency of late successional pre-colonisers and of non-opportunistic invading fungi. For the ascomycete pre-colonisers (*H. fragiforme* and *B. nummularia*) and the uncolonised control, there was a peak in the occurrence of invaders at 6 months and then a decline. The presence of cords attached to disks varied between pre-coloniser species and did not follow any pattern; the large number of cords on disks pre-colonised with *P. velutina* were produced by *P. velutina* itself. Unexpectedly, *P. impudicus* was rarely recovered by reisolation, which was likely to be due to the strain growing unexpectedly poorly during pre-colonisation.

The season in which disks were placed in the field (experiment A2) affected the retention of the early-successional species pre-colonisers, with much greater frequency of retention in autumn-released disks harvested after 6 months compared with spring-released disks (Table 2). The presence/absence of other pre-colonisers was unaffected by season of release, but there were differences in the occurrence of invading fungi depending on the harvest season, with higher occurrence in disks harvested in autumn. A similar pattern was found for the presence of attached cords. Staggering the release date of disks over 6 weeks had little effect on the retention of the original coloniser, or the occurrence of invading fungi (experiment C; Table 2).

Most of the species caused significant (*P*<0.05) wood decay during the 3-month pre-colonisation (Table 2), but significant further decay between 3 and 6 months was only found with *B. nummularia* and *P. velutina* (*P*<0.05). All species, except *T. versicolor*, effected significant (*P*<0.05) alteration of the pH of the wood after 3 months pre-colonisation, with the ascomycetes increasing the pH and the basidiomycetes decreasing it (Table 1). Retention of pre-colonisers decreased with increasing length of colonisation (3 or 6 months) before placement in the field (experiment B), with the exceptions of *P. velutina* where no change occurred, and *H. fragiforme* and *B. nummularia*, where there was no retention after 1 year. There was greater invasion of disks colonised by early-successional species after 3 months pre-colonisation than 6 months, whereas for late-successional species invasion was highest after 6 months pre-colonisation.

### Community profiles using isolation and direct incubation

Isolation of wood chips, cords and direct incubation of disks yielded 564 fungi, excluding pre-coloniser species (Table 3; Supplementary Table 5). These were grouped into 170 cOTUs based on ITS sequence (40.6%) and/or morphological similarity. Identification was not possible for many of the mycelia, owing to the inability to get mycelia into pure agar culture, insufficient material or unsuccessful DNA extraction or PCR. The most common invading cOTU (9.4% total cOTU occurrences) was obtained by disk incubation but did not occur in isolations onto agar; it was not possible to identify this highly black pigmented culture using ITS sequence due to repeated PCR failure. Although this culture was not identified microscopically, its mycelial morphology was similar to that of *Lasiosphaeris hispida*, the second-most dominant OTU identified by pyrosequencing (10.9% total sequences; Table 4; Supplementary Table 3). The other most commonly occurring cOTUs included *Hypocrea avellanea*, *Phanerochaete* sp. (likely *P. velutina*) and *Xenasmatella vaga* (Supplementary Table 5).

The number of cOTUs recovered was highest in control disks with no pre-colonisers, and lowest where pre-colonisers were fungi from late-successional stages (Table 3). Lower numbers of cOTUs were recovered after pre-colonisation by *S. hirsutum* relative to other secondary coloniser species. There were no significant differences in the number of cOTUs identified as Ascomycota (F_9,93_=0.481, *P*=0.84) or Basidiomycota (F_9,93_=1.363, *P*=0.216) recovered following different pre-coloniser species (Table 3). Significant differences in cOTU profiles following different pre-coloniser species were detected in pooled data across all experiments (F_9,93_=1.57, *P*<0.001; Supplementary Figure 3; Table 5). Pairwise testing showed significant (*P*<0.05) differences in community composition following certain pre-colonisers (Table 5). There were no significant differences (*P*>0.05) in the communities following uncolonised control disks or those pre-colonised with *H. fragiforme* or *B. nummularia*, but these were significantly (*P*<0.05) different from the community following pre-colonisation with *V. comedens*, *H. fasciculare* and *T. versicolor* (Table 5). However, a large amount of overlap between different communities makes overall patterns difficult to distinguish.

### Community profiles using metagenomics

**Figure 1:**
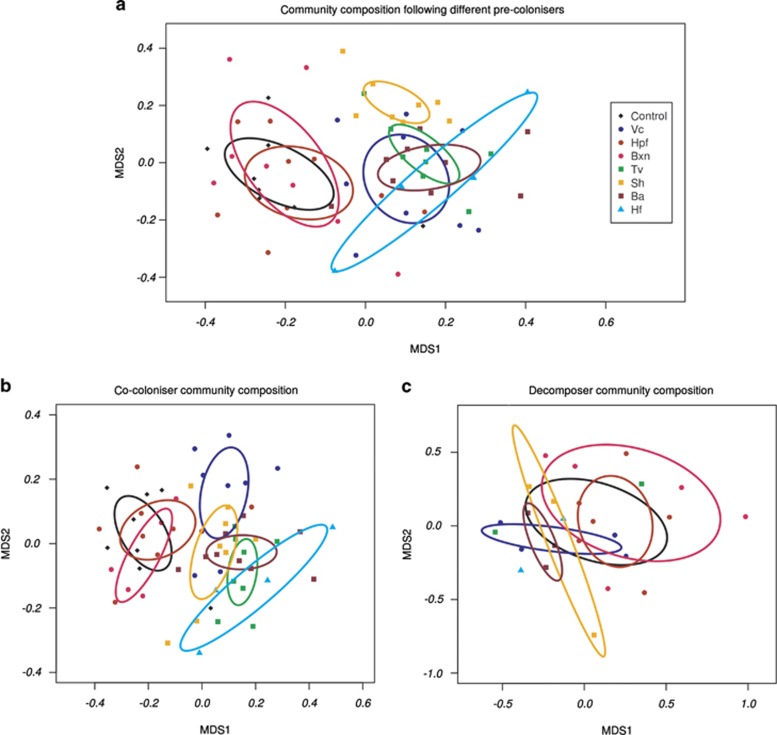
Fungal community composition in disks pre-colonised with different species, determined by pyrosequencing. (**a**) Non-metric multidimensional scaling (NMDS) plot (stress score 0.265) of fungal OTU (operational taxonomic units) composition based on the Bray–Curtis metric of dissimilarity, using all data. (**b**) NMDS plot (stress score 0.243) of fungal OTUs identified as ascomycetes (co-coloniser community; not known to have lignocellulolytic ability). (**c**) NMDS plot (stress score 0.149) of fungal OTUs identified as wood-decay basidiomycetes (decomposer community); samples containing <5% basidiomycete OTUs were excluded from the analysis. Points represent individual samples and ellipses indicate treatment (that is, pre-colonised by a particular species) means with 95% confidence intervals fitted onto the spatial ordination. Where ellipses are absent, insufficient sample numbers were present. See Table 1 for species name abbreviations.

There were no significant differences in variability of OTU profiles between different pre-coloniser species (Beta-disper; F_7,55_=0.5463, *P*=0.796; Supplementary Table 2), nor in diversity or community evenness (Table 4). However, community composition was significantly different between different pre-coloniser treatments (permutational multivariate ANOVA; F_7,55_=0.546, *P*<0.001; Figure 1a; Supplementary Table 2). Pairwise testing showed no significant differences (*P*>0.05) in community composition between disks pre-colonised with *H. fragiforme, B. nummularia* and uncolonised controls, but these three were significantly different (*P*<0.05) to all other pre-colonisers (with the exception of *H. fasciculare* versus *H. fragiforme*; Table 5). Community composition following pre-colonisation by *S. hirsutum* was significantly different (*P*<0.05) to all other treatments. However, there were no significant differences (*P*>0.05) between community composition following *V. comedens, T. versicolor, B. adusta* or *H. fasciculare.* No relationship was found between community composition versus disk area (Mantel's *r*=0.011, *P*=0.373), or volume (Mantel's *r*=0.03, *P*=0.204). Nor was there any correlation between community composition and position of the disk on the site (Mantel's *r*=0.044, *P*=0.073).

**Figure 2:**
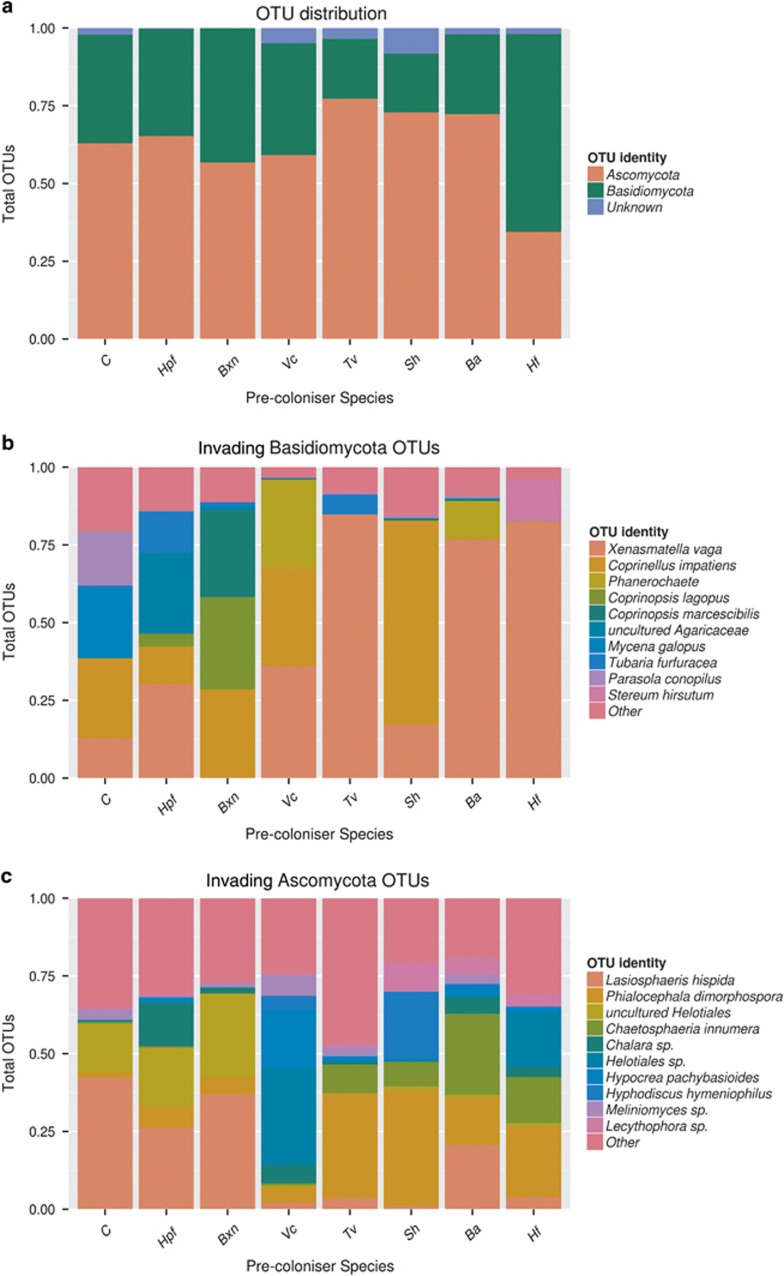
Composition of invading communities following different pre-colonisers. (**a**) The proportion of OTUs identified as Ascomycota, Basidiomycota or unknown. (**b**) The most frequent invasive OTUs identified as Basidiomycota. (**c**) The most frequent invasive OTUs identified as Ascomycota. There are no significant differences (F7,55=1.056, *P*=0.404) in the number of OTUs found between pre-coloniser treatments, or in the distribution of Ascomycota (F7,55=0.708, *P*=0.665) or Basidiomycota (F7,55=0.908, *P*=0.507). For **b** and **c**, OTUs comprising >10% of the total OTU count for a species were treated individually, and the remaining OTUs merged into the group 'other'.

Community profiles following all pre-colonisers were dominated by OTUs identified as ascomycetes (Figure 2a; Table 4). The OTUs responsible for causing the majority of the variation between community profiles included those identified as *Lasiosphaeris hispida*, *Xenasmatella vaga*, *Phialocephala dimorphospora*, *Coprinellus impatiens*, *Helotiales* spp. and *Chaetosphaeria innumera* (Figures 2b and c; Table 4; Supplementary Table 6). Different pre-colonisers affected the subsequent co-coloniser ascomycete communities, with those following control, *H. fragiforme* and *B. nummularia* significantly different to all other pre-coloniser species (F_7,51_=2.27, *P*<0.001; Beta-disper *P*=0.743; Figure 1b). However, there were no significant differences in the community composition of basidiomycete OTUs following different pre-colonisers (F_7,30_=1.11, *P*=0.257; Beta-disper *P*=0.09; Figure 1c).

## Discussion

Our use of metagenomic approaches to study assembly history in wood-decay communities clearly shows that priority effects are key determinants of fungal community development in beech wood. This was largely supported by both the culture-independent and traditional isolation approaches, although fewer species were detected by the latter and sometimes opportunistic ascomycete and zygomycete 'contaminants' dominated, which are usually considered to be of minor importance to the decay process (Lindner *et al.,* 2011). Two main groups of predecessor species—the ascomycete early-successional colonisers *H. fragiforme* and *B. nummularia*, and four basidiomycetes *V. comedens, T. versicolor, B. adusta* and *H. fasciculare*—resulted in communities distinct from each other. Further, pre-colonisation by the basidiomycete *S. hirsutum* resulted in a community distinct from all other predecessors, indicating that individual species may divert the pattern of succession. However, these differences reflect changes in ascomycete taxa—the secondary saprotrophs or 'co-coloniser' community—supposedly incapable of decomposing lignocellulose, which instead live opportunistically off other mycelia or products of their activity. These co-coloniser species may be more responsive to priority effects than decomposer species, perhaps because they are more sensitive to differences in resource alteration by different predecessors.

Priority effects are, at least partly, determined through biochemical alteration of the resource through enzyme activity, mycelial growth and deposition of secondary metabolites (Allison, 2012). Also, the alteration of wood pH may be highly significant in determining priority effects, as variations in pH affect fungal growth and decay rates, and could function as constitutive defence by inhibiting the growth of invasive species (Tudor *et al.,* 2013). Generally, wood-decay basidiomycetes prefer an acidic environment, whereas ascomycetes prefer slightly more alkaline conditions (Tudor *et al.,* 2013), which corresponds to the pH measurements in pre-colonised disks. *S. hirsutum* generated a significantly lower pH than all other pre-colonisers, which may be at least partly responsible for the different community and its defensive ability seen here and in other laboratory experiments (Hiscox *et al.,* 2015). Combative ability of the predecessor may partly explain the differences in subsequent community composition, as the ascomycete pre-colonisers are weaker combatants than the basidiomycete pre-colonisers (Hiscox *et al.*, 2015). However, combative abilities and life history strategies of the pre-coloniser basidiomycetes vary widely (Hiscox *et al.,* 2015), yet the composition of their successor communities were not significantly different. The mycelium of the primary coloniser is probably also an important resource for many secondary colonisers, especially those which derive their main source of nutrition directly from other mycelia (Lindahl and Finlay, 2006).

No differences in community composition were detected between disks pre-colonised by the ascomycetes (*H. fragiforme* and *B. nummularia*) and uncolonised controls; the ascomycete pre-coloniser and control disks led to a co-coloniser community distinct from that following all other pre-colonisers, but did not affect the subsequent decomposer community. This is consistent with previous findings that ascomycetes had no effect on subsequent colonisation of pine needles by basidiomycetes (Boberg *et al.*, 2014). However, both *H. fragiforme* and *B. nummularia* altered the resource during pre-colonisation, as evidenced by density loss (that is, decomposition), increased pH and pigment production; these alterations were clearly insufficient to drive changes in the community development relative to initially uncolonised, unaltered wood. The weak combative abilities of both *H. fragiforme* and *B. nummularia* may not have caused invasion of these disks to be significantly more challenging than the invasion of uncolonised resources, compared with the more antagonistic basidiomycete pre-colonisers (Hiscox *et al.*, 2015).

Replacement of the basidiomycete pre-colonisers within disks occurred progressively, and as expected the late-successional stage species lost far less territory than the early-successional species, reflecting their greater combative ability. Replacement of the ascomycete pre-colonisers, however, occurred very rapidly; the number of invading species peaked after 12 months and then decreased. This may have been a seasonal effect, or the result of an invading species inhibiting further colonisation by other invaders. The basidiomycete pre-coloniser species were still dominant after 12 months, and sometimes the pre-coloniser still accounted for over half of the OTUs recovered from the disk. The removal of OTUs corresponding to the pre-coloniser species prior to analyses was a conservative approach to avoid the extra variability the pre-coloniser would introduce, but this neglects the role of the pre-coloniser itself in community dynamics. There is a lot of variation in these communities, as successor species are interacting with the predecessor in a variety of ways (negatively, positively or neutrally). Further, there is considerable stochasticity, due, for example, to different pools of potential successor species arriving at each wood unit. Thus, modelling such multidimensional community patterns in a short experiment is difficult, and ideally, a longer study should be undertaken to reveal successional changes in wood-decay communities, after any predecessors have been fully replaced.

Environmental factors had an impact on assembly history: pre-coloniser retention and invasive species detection in samples put out in different seasons differed, indicating that environmental factors influence the ability of species to establish, either by affecting the outcomes of antagonistic interactions, or through effects on production and dispersal of spores (Edman *et al.,* 2004; Kauserud *et al.,* 2012). Many of the recovered disks were colonised by cord-forming fungi, in particular *Xenasmatella vaga*, *Phanerochaete* sp. and *H. fasciculare;* several individuals of these species were recovered from the site. Cord formation is an ability possessed almost exclusively by highly combative later secondary colonisers (Fricker *et al.,* 2008; Boddy *et al.,* 2009). Cord networks can cover large areas (many metres), and as such the presence of a few highly combative species on the field site may have masked certain priority effects. There will also be local variability in the presence of other potential invading species through differences in fruiting phenology and availability of different species of wood. For example, fungal colonisation patterns in *Picea abies we*re shown to differ between study areas, with species assemblages varying as much as 68% between different sites (Olsson *et al.,* 2011). It is, thus, important to use multiple sites in future studies of priority effects.

## Conclusions

Metagenomic and culture-based approaches revealed that distinct fungal communities occurred in beech disks following different pre-colonsiers. These communities differed in the composition of co-colonising ascomycetes but not of wood-decay basidiomycete species. The role of the co-coloniser component within the wood-decay community is not yet clear. Alteration of the resource by the pre-coloniser, especially changes in pH, is likely to drive the differences in assembly history, as are characteristics of the pre-coloniser, such as combative ability and qualitative differences in mycelial necromass (a resource for all invaders). Varying the season in which disks were put into the field led to differences in assembly history, especially with regard to the early-successional pre-colonisers. Future studies should cover a longer time to fully reveal patterns of succession in wood-decay communities, preferably across multiple sites with different potential successor communities, and should also include estimates of density to indicate extent of decay.

## Figures and Tables

**Table 1:** Details of species used

*Ecological role*	*Species*	*Abbreviation*	*Strain*		*Source*	*Initial pH*
Control	None	C	—	—	—	4.88^a^
Primary colonizer	*Hypoxylon fragiforme*	Hpf	HpfFF1	Ascomycota	Beech wood isolation	5.50^b^
	*Biscogniauxia nummularia*	Bxn	BxnFF1	Ascomycota	Beech wood isolation	5.31^b^
	*Vuilleminia comedens*	Vc	VcWVJH1	Basidiomycota	Beech wood isolation	3.67^c^
Early secondary coloniser	*Trametes versicolor*	Tv	TvCCJH1	Basidiomycota	Fruit body isolation	4.83^a^
	*Stereum hirsutum*	Sh	ShSS1	Basidiomycota	Fruit body isolation	3.31^d^
	*Bjerkandera adusta*	Ba	BaSS1	Basidiomycota	Fruit body isolation	4.16^e^
Late secondary/tertiary coloniser	*Hypholoma fasciculare*	Hf	HfDD3	Basidiomycota	Fruit body isolation	3.83^c^
	*Phanerochaete velutina*	Pv	Pv29	Basidiomycota	Beech wood isolation	3.88^ce^
	*Phallus impudicus*	Pi	PiJHY4	Basidiomycota	Cord isolation	—

Initial wood pH was determined using pre-colonised wood; different superscript letters indicate significant (*P*<0.05) differences in pH between pre-coloniser species.

**Table 2:** Occurrence of original pre-coloniser, invading fungi and attached cords from disks over experimental treatments A–C

*Experiment*	*Pre-coloniser*	*Pre-col time (mo)*	*Density (g cm*^−*3*^)	*Release season*	*% Disks*											
					*Original pre-coloniser*				*Invading fungi*				*Attached cords*			
				*Months*	*6*	*12*	*18*	*24*	*6*	*12*	*18*	*24*	*6*	*12*	*18*	*24*
*A1*																
Effect of time in field		3	0.486	Sept 2011	—	—	—	—	80	90	70	50	60	30	40	12.5
	*V. comedens*^a^	3	0.481	Sept 2011	81.8	18.2	0	0	45.5	63.6	77.8	80	9.1	45.5	33.3	20
	*H. fragiforme*^a^	3	0.444^b^	Sept 2011	22.2	0	0	0	66.7	90	66.7	66.7	44.4	30	11.1	11.1
	*B. nummularia*^a^	3	0.473b,^c^	Sept 2011	50	0	0	0	100	80	80	50	37.5	40	40	37.5
	*T. versicolor*^a^	3	0.443^b^	Sept 2011	100	80	80	9.1	22.2	80	100	72.7	0	30	20	18.2
	*S. hirsutum*^a^	3	0.450^b^	Sept 2011	90	88.9	60	80	40	22.2	90	80	10	11.1	10	60
	*B. adusta*^a^	3	0.460^b^	Sept 2011	100	60	40	0	33.3	60	80	88.9	0	50	20	22.2
	*H. fasciculare*^a^	3	0.469^b^	Sept 2011	100	80	85.7	57.1	11.1	80	100	100	33.3	50	14.3	71.4
	*P. velutina*	3	0.481^b^	Sept 2011	100	90	66.7	50	0	20	11.1	50	100	70	55.6	16.7
	*P. impudicus*	3	—	Sept 2011	0	0	0	0	66.7	90	50	90	44.4	40	20	40
																
*A2*																
Effect of season of placement in the field	Control	3	—	Dec 2011	—	—	—	—	100	83.3	—	—	11.1	16.7	—	—
	*V. comedens*	3	—	Dec 2011	40	0	—	—	100	100	—	—	0	22.2	—	—
	*T. versicolor*	3	—	Dec 2011	100	85.7	—	—	100	85.7	—	—	20	0	—	—
	*H. fasciculare*	3	—	Dec 2011	100	100	—	—	100	100	—	—	11.1	66.7	—	—
	Control	3	—	Mar 2012	—	—	—	—	50	75	—	—	50	62.5	—	—
	*V. comedens*	3	—	Mar 2012	20	0	—	—	60	33.3	—	—	40	16.7	—	—
	*H. fragiforme*	3	—	Mar 2012	11.1	0	—	—	88.9	42.9	—	—	22.2	0	—	—
	*B. nummularia*	3	—	Mar 2012	11.1	0	—	—	77.8	50	—	—	44.4	25	—	—
	*T. versicolor*	3	—	Mar 2012	100	77.8	—	—	55.6	66.7	—	—	11.1	11.1	—	—
	*S. hirsutum*	3	—	Mar 2012	88.9	71.4	—	—	55.6	71.4	—	—	22.2	42.9	—	—
	*B. adusta*	3	—	Mar 2012	90	75	—	—	40	62.5	—	—	30	25	—	—
	*H. fasciculare*	3	—	Mar 2012	100	100	—	—	42.9	100	—	—	28.6	25	—	—
	*P. velutina*	3	—	Mar 2012	100	100	—	—	12.5	100	—	—	100	100	—	—
	*P. impudicus*	3	—	Mar 2012	0	0	—	—	66.7	66.7	—	—	33.3	55.6	—	—
	Control	3	—	Jun 2012	—	—	—	—	100	100	—	—	25	37.5	—	—
	*V. comedens*	3	—	Jun 2012	25	0	—	—	100	25	—	—	25	0	—	—
	*T. versicolor*	3	—	Jun 2012	66.7	66.7	—	—	77.8	100	—	—	22.2	0	—	—
	*H. fasciculare*	3	—	Jun 2012	100	100	—	—	100	100	—	—	100	0	—	—
																
*B*																
** **Effect of length of pre-colonisation	Control	3	—	Sept 2012	—	—	—	—	—	—	—	—	—	—	—	—
	*V. comedens*	6	0.457^a^	Sept 2012	—	—	—	—	—	—	—	—	—	—	—	—
	*H. fragiforme*	6	0.440	Sept 2012	—	—	—	—	—	—	—	—	—	—	—	—
	*B. nummularia*	6	0.430b,^c^	Sept 2012	—	—	—	—	—	—	—	—	—	—	—	—
	*T. versicolor*	6	0.408^b^	Sept 2012	—	—	—	—	—	—	—	—	—	—	—	—
	*S. hirsutum*	6	0.449^b^	Sept 2012	—	—	—	—	—	—	—	—	—	—	—	—
	*B. adusta*	6	0.428^b^	Sept 2012	—	—	—	—	—	—	—	—	—	—	—	—
	*P. velutina*	6	0.440b,^c^	Sept 2012	—	—	—	—	—	—	—	—	—	—	—	—
																
*C*																
** **Effect of short-term variation in field placement date	Control	3	—	Sept 2011 +2 wks	—	—	—	—	80	—	—	—	10	—	—	—
	Control	3	—	Sept 2011 +4 wks	—	—	—	—	80	—	—	—	30	—	—	—
	Control	3	—	Sept 2011 +6 wks	—	—	—	—	44.4	—	—	—	11.1	—	—	—
	*V. comedens*	3	—	Sept 2011 +2 wks	100	—	—	—	0	—	—	—	0	—	—	—
	*V. comedens*	3	—	Sept 2011 +4 wks	100	—	—	—	0	—	—	—	0	—	—	—
	*V. comedens*	3	—	Sept 2011 +6 wks	63.6	—	—	—	36.4	—	—	—	36.4	—	—	—
	*T. versicolor*	3	—	Sept 2011 +2 wks	100	—	—	—	11.1	—	—	—	0	—	—	—
	*T. versicolor*	3	—	Sept 2011 +4 wks	100	—	—	—	0	—	—	—	20	—	—	—
	*T. versicolor*	3	—	Sept 2011 +6 wks	90	—	—	—	20	—	—	—	10	—	—	—
	*H. fasciculare*	3	—	Sept 2011 +2 wks	100	—	—	—	10	—	—	—	70	—	—	—
	*H. fasciculare*	3	—	Sept 2011 +4 wks	100	—	—	—	25	—	—	—	100	—	—	—
	*H. fasciculare*	3	—	Sept 2011 +6 wks	100	—	—	—	0	—	—	—	66.7	—	—	—

Abbreviations: mo, months; wks, weeks.

Nearly all disks pre-colonised with *H. fasciculare* set out after September 2012 disappeared, although disks colonised by other species set out at the same time were recovered; this selective removal was presumed to be due to mammal activity. Data are shown as graphs in Supplementary Figures 2A and E.

a Samples used for DNA extraction and pyrosequencing (12-month harvest)

b Density of colonised disks were significantly (*P*<0.05) different from density of uncolonised control disks

c Density of disks after 6 months colonisation was significantly (*P*<0.05) lower (that is, more decayed) than after 3 months.

**Table 3:** Summary cOTU (cultured operational taxonomic unit) statistics by different pre-colonisers

		*Pre-coloniser*									
		*Control*	V. comedens	H. fragiforme	Biscogniauxia sp.	T. versicolor	S. hirsutum	B. adusta	H. fasciculare	P. velutina	P. impudicus
Replicates included in analysis		16	15	9	9	16	8	8	8	7	7
Number counts		119	98	65	68	57	29	52	20	10	46
No. cOTUs	Total	51	46	42	48	36	21	34	16	10	32
	Mean counts per sample	7.44	6.53	7.22	7.56	3.56	3.63	6.50	2.50	2.14	6.57
	Mean cOTUs per sample	3.2	3.1	4.7	5.3	2.3	2.6	4.3	2.0	1.4	4.6
Ascomycota	No.	37	27	21	24	20	13	21	7	6	19
	% Total cOTUs	72.5	58.7	50.0	50.0	55.6	61.9	61.8	43.8	60.0	59.4
	% Total counts	31.1	27.6	32.3	35.3	35.1	44.8	40.4	35.0	60.0	41.3
Basidiomycota	No.	5	12	15	19	8	1	8	8	3	4
	% Total cOTUs	9.8	26.1	35.7	39.6	22.2	4.8	23.5	50.0	30.0	12.5
	% Total counts	4.2	12.2	23.1	27.9	14.0	3.4	15.4	40.0	30.0	8.7
Unidentified	No.	9	7	6	5	8	7	5	1	1	9
	% Total cOTUs	17.6	15.2	14.3	10.4	22.2	33.3	14.7	6.3	10.0	28.1
	% Total counts	7.6	7.1	9.2	7.4	14.0	24.1	9.6	5.0	10.0	19.6
Dominant replacing cOTU	Name	G16	*Hypocrea avellanea*	G16	G16	G35	*Hypocrea avellanea*	*Absidia glauca*	G14	*Mortierella* sp.	G16
	% Total cOTU counts	17.0	23.0	14.0	13.0	14.0	17.0	10.0	10.0	10	11
	Asco/basidiomycota	Asco^a^	Asco^a^	Asco^a^	Asco^a^	—	Asco	Asco	—	Asco	Asco^a^
*H*	(F_9,93_=15.7, *P*<0.001)	6.119	2.423	2.659	2.394	0.965	0.900	1.698	0.711	0.517	1.606
*α*	(F_9,93_=60.9, *P*<0.001)	0.774	0.465	0.372	0.330	0.200	0.190	0.206	0.124	0.114	0.176
*E*	(F_9,93_=27.9, *P*<0.001)	1.430	0.830	0.741	0.652	0.278	0.242	0.391	0.153	0.118	0.335

Abbreviation: ANOVA, analysis of variance.

Results from one-way ANOVA comparisons of Shannon diversity (*H*), Fishers' alpha (*α*) and Pielou's evenness (*E*) are given as the F-statistic with degrees of freedom and *P*-value.

a Phylum is based on mycelial characteristics of unknown fungus G16.

**Table 4:** Summary OTU statistics

		*Pre-coloniser*							
		*Control*	V. comedens	H. fragiforme	Biscogniauxia sp.	T. versicolor	S. hirsutum	B. adusta	H. fasciculare
Replicates included in analysis		8	9	9	8	8	8	9	4
No. OTUs pre-rarefaction (including pre-coloniser)	Total	373	299	332	272	235	170	200	150
	Mean counts per sample	6149	4194	5339	4421	3785	1545	3433	2558
	Mean OTUs per sample	79	61	65	53	50	40	45	48
No. OTUs after rarefaction (inlcuding pre-coloniser)	Total	88	71	79	64	58	58	58	42
	Mean counts per sample	205.5	205.9	205.4	206.3	206.4	206.6	206.7	206.3
	Mean OTUs per sample	19.3	16.6	16.7	11.0	15.0	14.1	15.0	14.5
No. OTUs pre-rarefaction (excluding pre-coloniser)	Total	373	298	329	273	231	165	196	147
	Mean counts per sample	6149	4058	5234	4421	3008	783	2479	3869
	Mean OTUs per sample	79	60	64	53	49	39	43	67
No. OTUs after rarefaction (excluding pre-coloniser)	Total	88	70	78	64	57	57	57	41
	Mean counts per sample	205.5	175.9	192.4	206.3	111.1	89.0	142.9	113.5
	Mean OTUs per sample	19.3	15.9	16.1	11.0	14.1	13.1	14.1	14.0
Pre-coloniser present?	Y/N	—	Y	Y	N	Y	Y	Y	Y
	% total OTU counts^a^	—	14.6	6.3	0	46.15	56.93	30.68	45
Ascomycota	No.	62	53	57	43	39	37	35	27
	% total OTUs	70.5	75.7	73.1	67.2	68.4	64.9	61.4	65.9
	% total counts	62.9	18.5	65.2	56.8	77.3	72.9	72.3	34.1
Basidiomycota	No.	21	12	19	19	12	16	18	10
	% total OTUs	23.9	17.1	24.4	29.7	21.1	28.1	31.6	24.4
	% total counts	34.9	59.3	34.5	43.1	19.2	18.8	25.7	63.9
Unidentified	No.	5	5	2	2	6	4	4	4
	% total OTUs	5.7	7.1	2.6	3.1	10.5	7.0	7.0	9.8
	% total counts	2.3	4.7	0.3	0.1	3.5	8.3	2.0	2.0
Dominant replacing OTU	Name	*Lasiosphaeris hispida*	*Helotiales* sp.	*Lasiosphaeris hispida*	*Lasiosphaeris hispida*	*Phialocephala dimorphospora*	*Phialocephala dimorphospora*	*Xenasmatella vaga*	*Xenasmatella vaga*
	% total OTU counts	26.6	18.5	18.8	21.0	26.1	27.0	19.7	52.4
	Asco/basidiomycota	Ascomycota	Ascomycota	Ascomycota	Ascomycota	Ascomycota	Ascomycota	Basidiomycota	Basidiomycota
Shannon diversity (*H*)	(F_7,55_=1.3, *P*=0.28)	1.668	1.395	1.518	0.925	1.843	1.637	1.625	1.539
Fisher's alpha (*α*)	(F_7,55_=0.9, *P*=0.51)	5.379	4.456	4.394	2.673	5.722	5.085	4.532	4.675
Pielou's evenness (*E*)	(F_7,55_=2.1, *P*=0.06)	0.558	0.517	0.541	0.352	0.693	0.663	0.624	0.660

Abbreviations: ANOVA, analysis of variance; N, no; OTU, operational taxonomic unit; Y, yes.

Number of OTUs pre- and post rarefaction are given for data including and excluding OTUs corresponding to the pre-coloniser species; where there was no pre-coloniser (control) or the pre-coloniser was absent in the OTU profile (*Biscogniauxia sp*.), data are the same in both instances.

All other analyses were performed on data with pre-coloniser OTUs excluded. Results from one-way ANOVA comparisons of Shannon diversity (*H*), Fishers' alpha (*α*) and Pielou's evenness (*E*) are given as degrees of freedom (df), the F-statistic and *P*-value; no significant differences (*P*>0.05) were found in any of these measures between different pre-colonisers.

a This corresponds to total OTU counts including the pre-coloniser species.

**Table 5:** Pairwise differences in community profiles following different pre-coloniser treatments using metagenomics (OTUs) vs traditional culture (cOTU) techniques

*Pairwise comparison*	*OTU profiles*			*cOTU profiles*		
	*df*	*F*	P *(adj)*	*df*	*F*	P *(adj)*
*Control–V. comedens*	16	2.510	0.014^a^	30	2.660	0.009^a^
*Control–H. fragiforme*	16	0.840	0.654	24	1.050	0.500
*Control–B. nummularia*	15	0.749	0.750	24	1.050	0.500
*Control–T. versicolor*	15	2.786	0.011^a^	31	3.070	0.009^a^
*Control–S. hirsutum*	15	3.545	0.011^a^	23	1.690	0.068
*Control–B. adusta*	16	2.839	0.012^a^	23	1.370	0.210
*Control–H. fasciculare*	11	2.193	0.026^a^	23	2.840	0.009^a^
*Control–P. velutina*	—	—	—	22	2.170	0.017^a^
*Control–P. impudicus*	—	—	—	22	0.910	0.721
*V. comedens–H. fragiforme*	17	1.844	0.031^a^	23	2.270	0.031^a^
*V. comedens–B. nummularia*	16	2.058	0.018^a^	23	2.060	0.035^a^
*V. comedens–T. versicolor*	16	1.818	0.022^a^	30	2.240	0.009^a^
*V. comedens–S. hirsutum*	16	2.377	0.011^a^	22	0.970	0.633
*V. comedens–B. adusta*	17	1.874	0.031^a^	22	1.830	0.061
*V. comedens–H. fasciculare*	12	1.412	0.144	22	2.290	0.02^a^
*V. comedens–P. velutina*	—	—	—	21	1.710	0.061
*V. comedens–P. impudicus*	—	—	—	21	2.050	0.042^a^
*H. fragiforme–B. nummularia*	16	0.661	0.873	17	0.740	0.930
*H. fragiforme–T. versicolor*	16	1.961	0.019^a^	24	2.160	0.009^a^
*H. fragiforme–S. hirsutum*	16	2.600	0.011^a^	16	1.420	0.103
*H. fragiforme–B. adusta*	17	2.029	0.052	16	1.180	0.340
*H. fragiforme–H. fasciculare*	12	1.553	0.120	16	2.040	0.015^a^
*H. fragiforme–P. velutina*	—	—	—	15	1.720	0.025^a^
*H. fragiforme–P. impudicus*	—	—	—	15	0.830	0.873
*B. nummularia–T. versicolor*	15	2.148	0.011^a^	24	1.750	0.03^a^
*B. nummularia–S. hirsutum*	15	2.675	0.012^a^	16	0.890	0.796
*B. nummularia–B. adusta*	16	2.432	0.016^a^	16	0.770	0.931
*B. nummularia–H. fasciculare*	11	1.875	0.017^a^	16	1.530	0.048^a^
*B. nummularia–P. velutina*	—	—	—	15	1.310	0.070
*B. nummularia–P. impudicus*	—	—	—	15	0.540	0.992
*T. versicolor–S. hirsutum*	15	1.439	0.017^a^	23	1.240	0.217
*T. versicolor–B. adusta*	16	1.439	0.144	23	1.570	0.066
*T. versicolor–H. fasciculare*	11	1.137	0.356	23	1.580	0.050
*T. versicolor–P. velutina*	—	—	—	22	0.780	0.931
*T. versicolor–P. impudicus*	—	—	—	22	1.820	0.0225^a^
*S. hirsutum–B. adusta*	16	2.175	0.019^a^	15	0.760	0.931
*S. hirsutum–H. fasciculare*	11	1.933	0.026^a^	15	1.290	0.204
*S. hirsutum–P. velutina*	—	—	—	14	0.800	0.931
*S. hirsutum–P. impudicus*	—	—	—	14	0.930	0.723
*B. adusta–H. fasciculare*	12	1.236	0.306	15	1.560	0.045^a^
*B. adusta–P. velutina*	—	—	—	14	0.980	0.721
*B. adusta–P. impudicus*	—	—	—	14	0.590	0.963
*H. fasciculare–P. velutina*	—	—	—	14	1.220	0.273
*H. fasciculare–P. impudicus*	—	—	—	14	1.840	0.0168^a^
*P. velutina–P. impudicus*	—	—	—	13	1.360	0.096

Abbreviations: df, degrees of freedom; PERMANOVA, permutational multivariate analysis of variance.

Comparisons were performed using Adonis/PERMANOVA, following which a *P*-adjustment was performed to reduce the effect of false discovery rates (Benjamini and Hochberg, 1995); adjusted *P*-values are shown.

a Significant difference in community composition (*P*<0.05).
