# Supplementary material for: Priority effects during fungal community establishment in beech wood
Source: ISME J. 2015 Mar 20;9(10):2246–60. doi: 10.1038/ismej.2015.38 (PMC4579477; doi:10.1038/ismej.2015.38)
Supplement: Supplementary Figure 1 [file ismej201538x1.pdf]

	1	2	3	4	5	6	7	8	9	10
1	Ba	Pv	Control	Pi	Hf	Bxn	Tv	Vc	Sh	Hpf
2	Hpf	Tv	Pi	Control	Vc	Sh	Pv	Hf	Bxn	Ba
3	Hf	Bxn	Hpf	Ba	Tv	Control	Sh	Pv	Pi	Vc
4	Pi	Vc	Bxn	Sh	Ba	Pv	Hf	Hpf	Tv	Control
5	Sh	Hpf	Pv	Tv	Pi	Vc	Ba	Control	Hf	Bxn
6	Vc	Sh	Ba	Hpf	Pv	Pi	Bxn	Tv	Control	Hf
7	Control	Hf	Sh	Bxn	Hpf	Tv	Vc	Ba	Pv	Pi
8	Tv	Control	Vc	Hf	Sh	Hpf	Pi	Bxn	Ba	Pv
9	Pv	Pi	Hf	Vc	Bxn	Ba	Control	Sh	Hpf	Tv
10	Bxn	Ba	Tv	Pv	Control	Hf	Hpf	Pi	Vc	Sh

←2.5m→
