# Supplementary material for: Priority effects during fungal community establishment in beech wood
Source: ISME J. 2015 Mar 20;9(10):2246–60. doi: 10.1038/ismej.2015.38 (PMC4579477; doi:10.1038/ismej.2015.38)

Supplementary Figure 2A

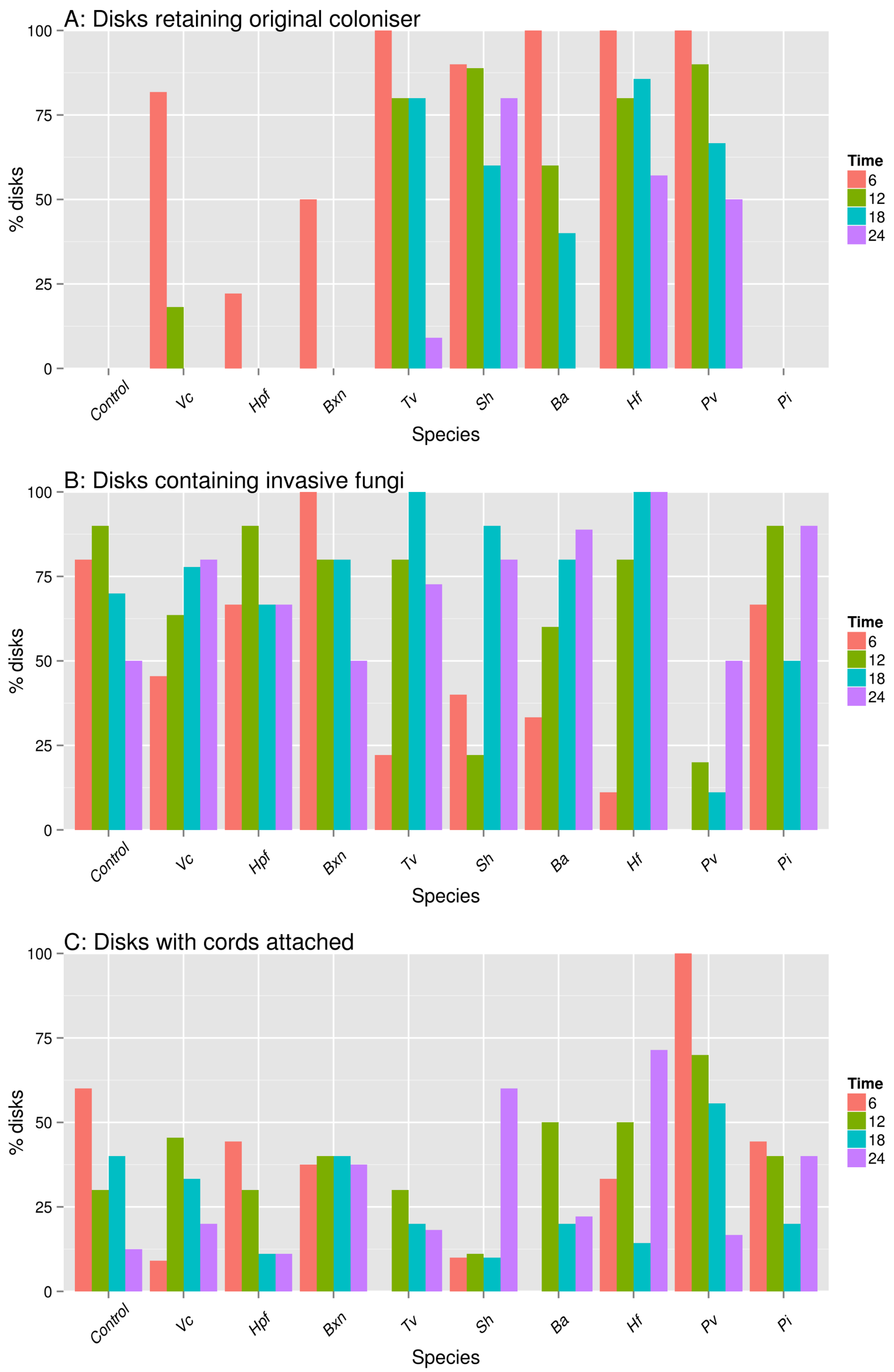

Supplementary Figure 2B

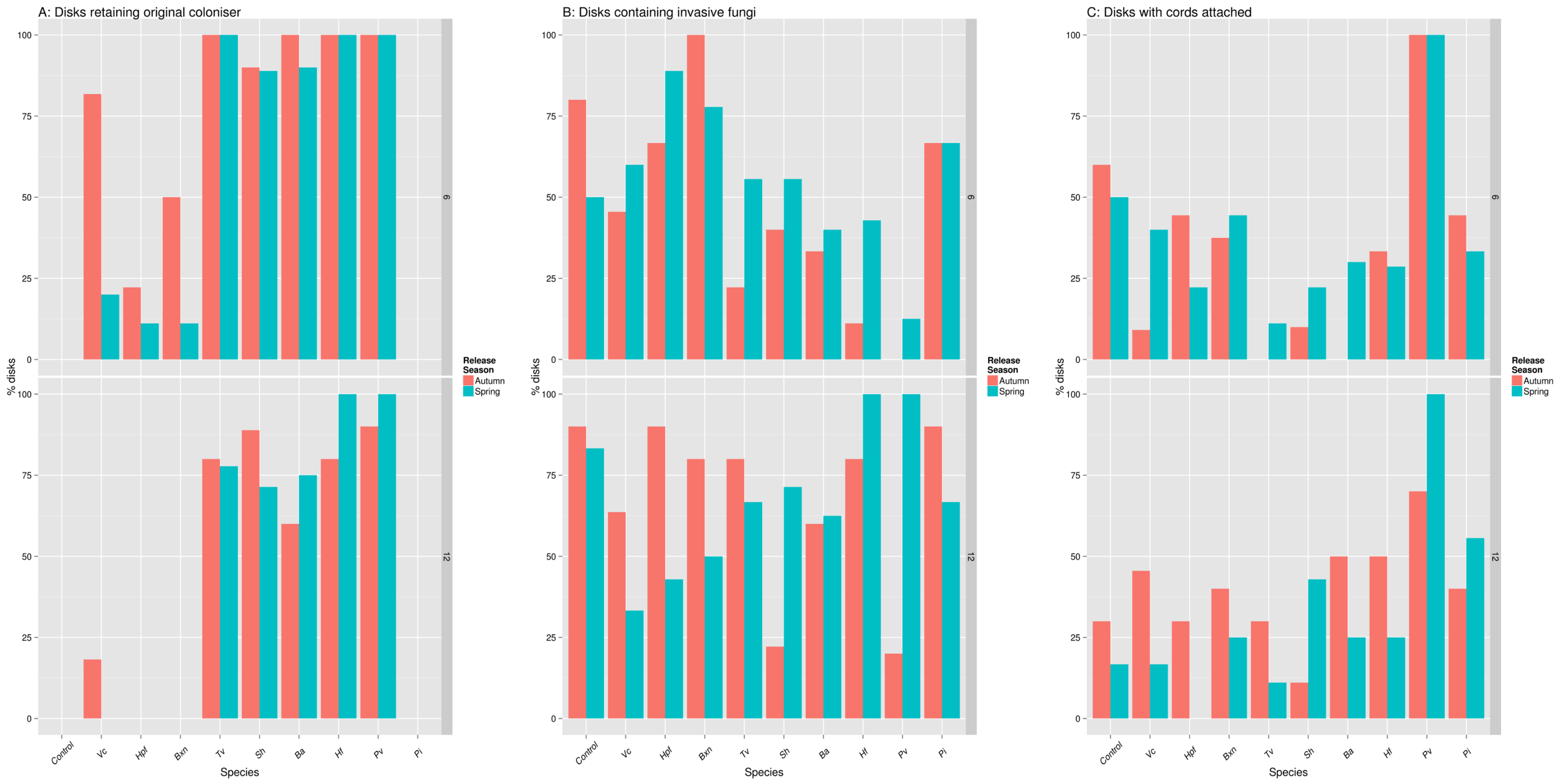

Supplementary Figure 2C

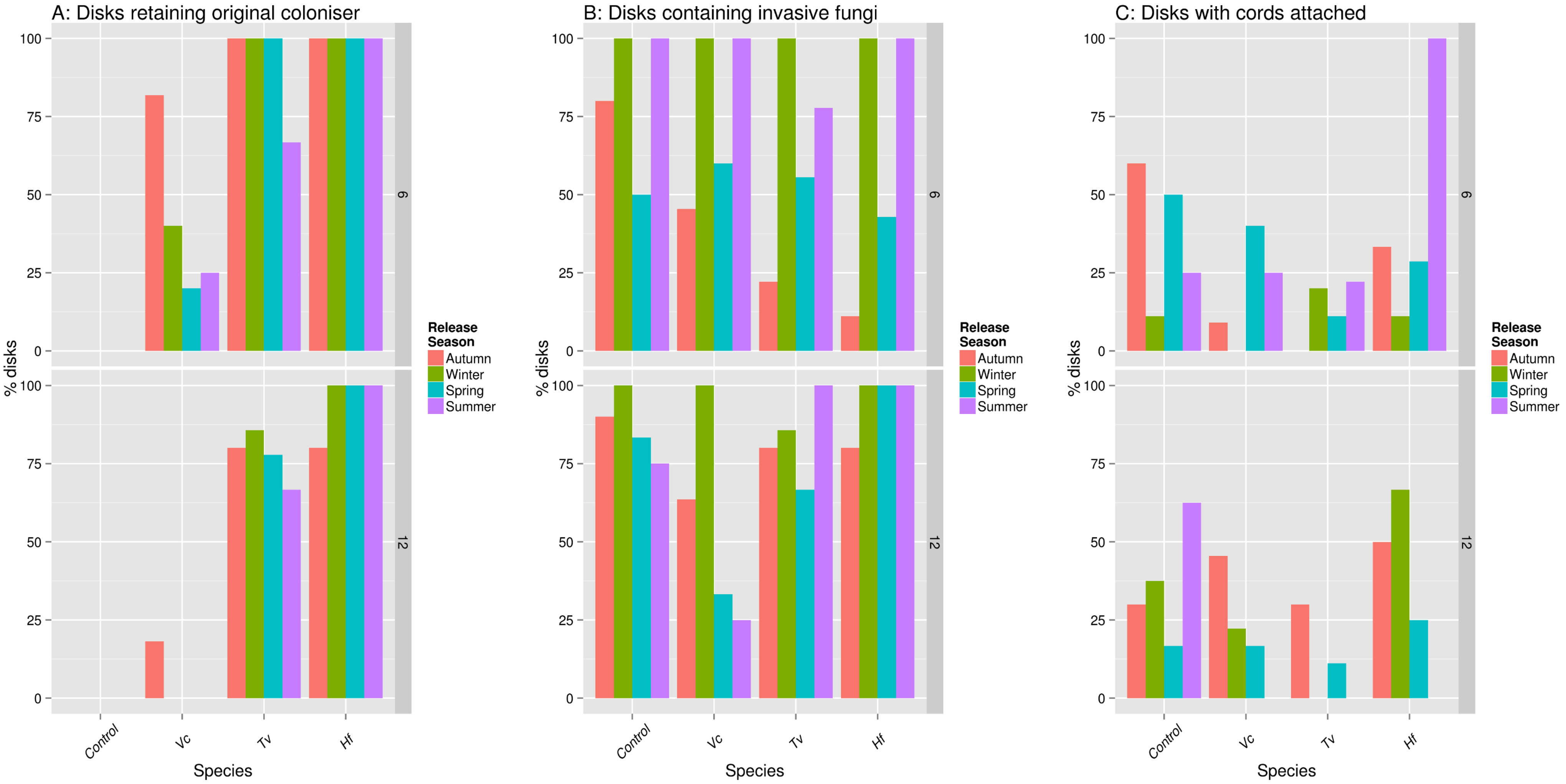

Supplementary Figure 2D

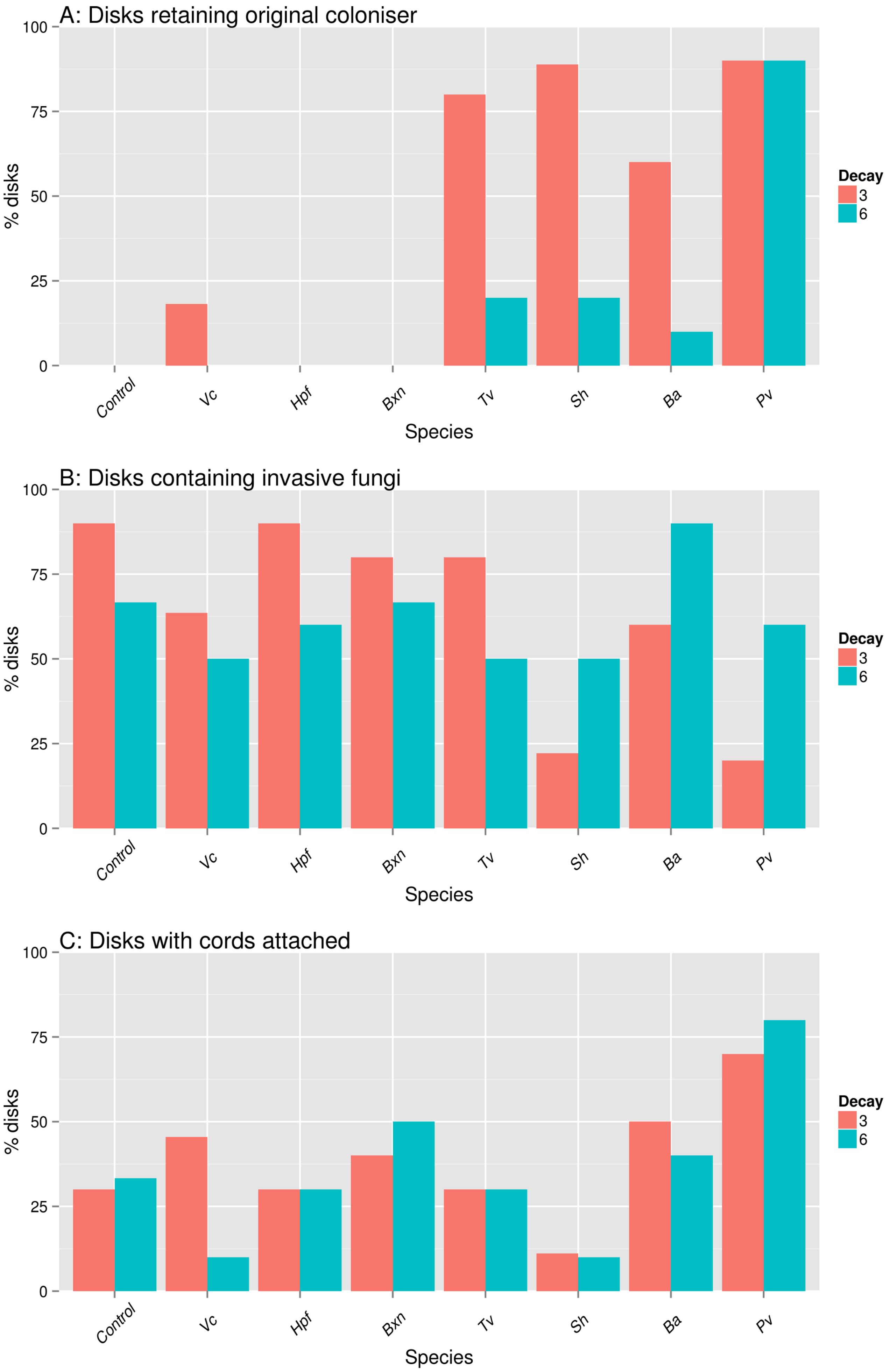

Supplementary Figure 2E

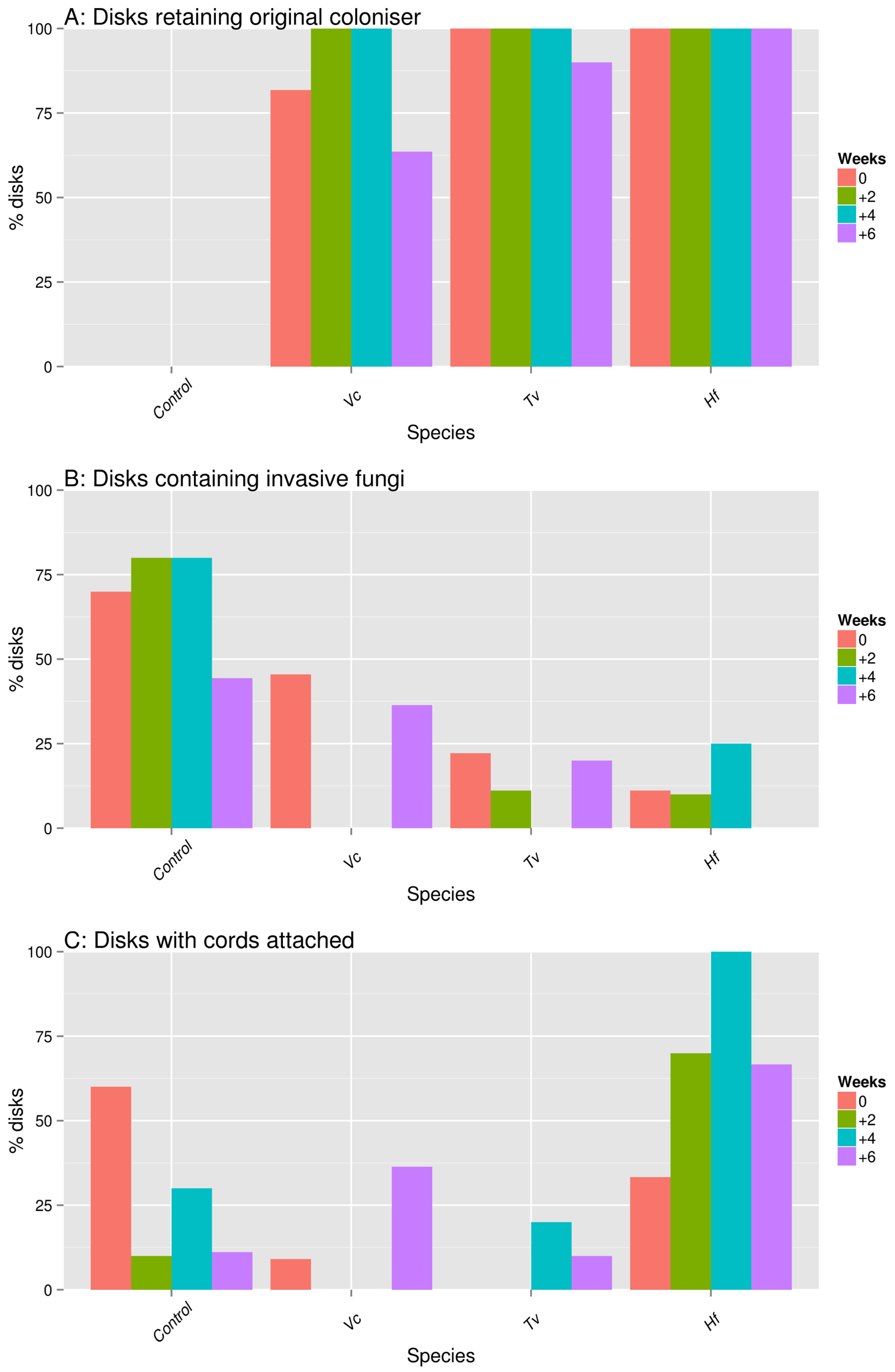

Supplement: Supplementary Figure 2 [file ismej201538x2.pdf]
