# Supplementary figures and images for: Priority effects during fungal community establishment in beech wood
Source: ISME J. 2015 Mar 20;9(10):2246–60. doi: 10.1038/ismej.2015.38 (PMC4579477; doi:10.1038/ismej.2015.38)

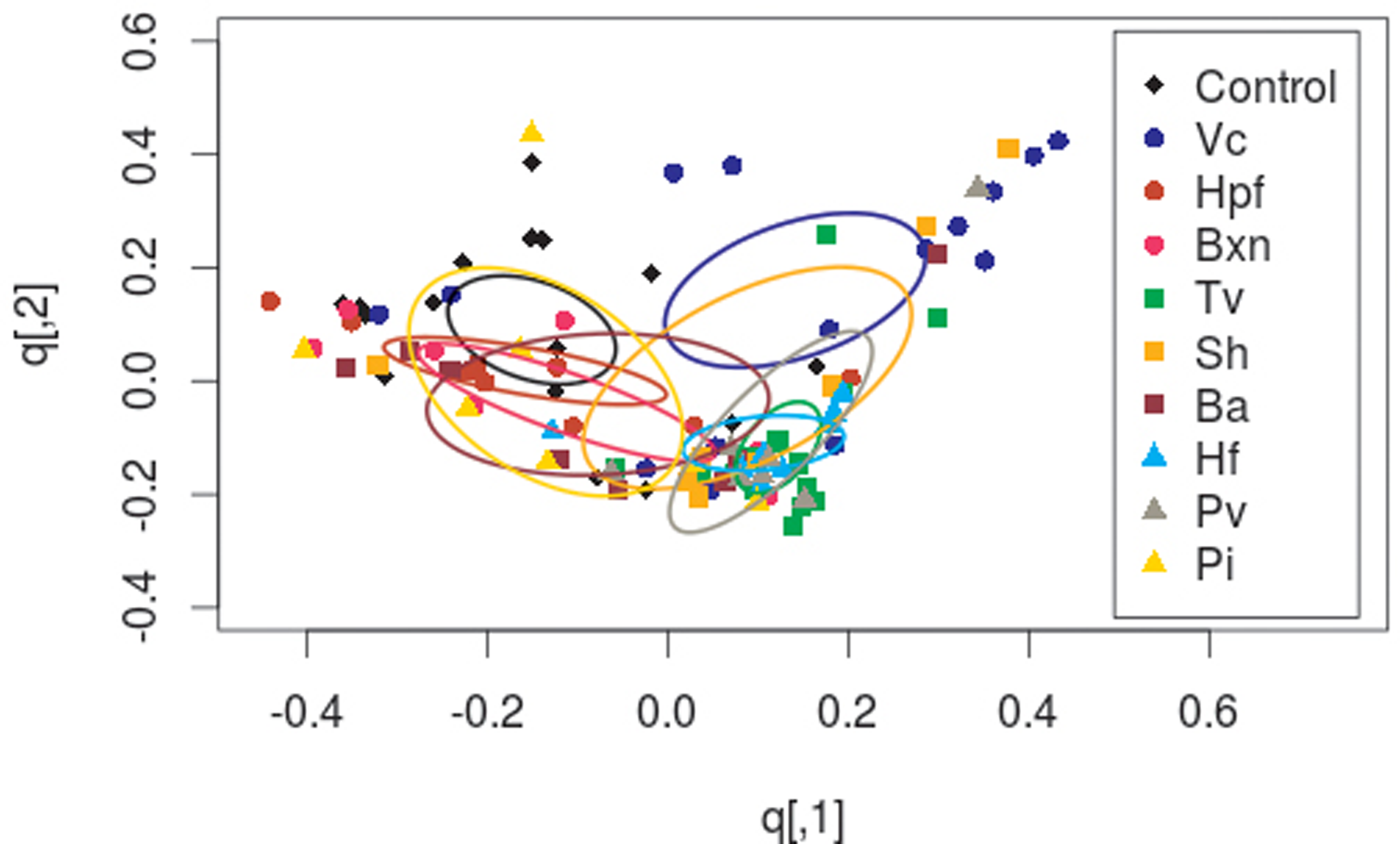

Supplement: Supplementary Figure 3 [file ismej201538x3.tif]
