# Supplementary material for: Priority effects during fungal community establishment in beech wood
Source: ISME J. 2015 Mar 20;9(10):2246–60. doi: 10.1038/ismej.2015.38 (PMC4579477; doi:10.1038/ismej.2015.38)
Supplement: Supplementary Figure Legends [file ismej201538x4.doc]

**Supplementary Figure Legends**

**Supplementary Figure 1.** Distribution of samples on the field site. Disks were placed in the centre of each grid square, in a randomised control block design (Latin square). Grey squares indicate location of temperature recorders. See Table 1 for species name abbreviations.

**Supplementary Figure 2.** Changes in community composition within disks, detailing the number of disks that A: retain the original pre-coloniser, B: contain invasive fungi, and C: have mycelial cords attached.

**2A:** Changes in community composition over the 24 month experimental period (Time) following release of disks into the field in September 2011. **2B:** Changes in community composition within disks released into the field seasonally after 6 and 12 months (experiment A2). **2C:** Changes in community composition within disks released into the field in autumn and spring after 6 and 12 months (experiment A2). **2D:** Changes in community composition within disks that had staggered release into the field over 6 weeks (experiment C). **2E:** Changes in community composition within disks that had been pre-colonised for either 3 or 6 months prior to release into the field (experiment B).

**Supplementary Figure 3.** Fungal community composition in disks pre-colonised with different species, determined by traditional community profiling techniques. The figure shows an ordination of fungal cOTU (cultured operational taxonomic units) composition based on classical multidimensional scaling using the Bray-Curtis metric of dissimilarity. Points represent individual samples and ellipses indicate treatment (species) means with 95% confidence intervals fitted onto the spatial ordination. See Table 1 for species name abbreviations.
