# Supplementary material for: Priority effects during fungal community establishment in beech wood
Source: ISME J. 2015 Mar 20;9(10):2246–60. doi: 10.1038/ismej.2015.38 (PMC4579477; doi:10.1038/ismej.2015.38)
Supplement: Supplementary Table 3 [file ismej201538x7.pdf]

Supplementary Table 3. Occurrence and identification of OTUs by precoloniser species. Each OTU is given as a % of the total OTUs detected across all replicates of each precoloniser species. The sum occurrence across all precoloniser species gives a measure of the overall abundance of each OTU. OTU identity was determined using massBLASTer, and the taxon with the best e-value was selected, and the relevant accession number is given. Taxonomy was determined by Galaxy454. -, not detected or data unavailable. OTUs were divided into one of two groups based on their ecological role: those that are known to directly contribute to wood decomposition were assigned to the decomposer group (D), whereas those that are secondary saprotrophs (live off exudates or the mycelium of wood decomposers) assigned to the co-coloniser group (C). Broadly, the Ascomycota were group C and the Basidiomycota group D.

OTU ID no.	Occurrence as % of total sequences occurring in each precoloniser species								Average occurrence	Identification details			Taxonomy			Functional group
	Control	*V.comedens*	*H. fragiforme*	*B. nummularia*	*T. versicolor*	*S. hirsutum*	*B. adusta*	*H. fasciculare*		Taxon name	Accession #	E-value	Phylum	Subphylum	Class	
priority_effects_CL_4	4.44	12.93	10.39	0.06	16.31	3.23	19.67	52.42	14.93	*Xenasmatella vaga*	UDB000519	2E-131	Basidiomycota	n	Agaricomycetes	D
priority_effects_CL_9	8.94	11.29	4.21	12.18	-	12.36	0.08	-	8.18	*Coprinellus impatiens*	JN943130	1e-153	Basidiomycota	n	Agaricomycetes	D
priority_effects_CL_13	-	10.28	-	-	-	-	3.11	0.22	4.54	*Phanerochaete sp.*	UDB017837	1E-153	Basidiomycota	n	n	D
priority_effects_CL_34	7.73	-	-	0.18	-	-	-	-	3.95	*Mycena galopus*	JQ926166	2E-156	Basidiomycota	n	Agaricomycetes	D
priority_effects_CL_39	-	-	-	3.88	-	-	-	-	3.88	uncultured Basidiomycete	KF297103	7e-120	Basidiomycota	n	Agaricomycetes	D
priority_effects_CL_15	0.06	-	1.44	12.79	-	-	0.00	-	3.57	*Coprinopsis lagopus*	HM126487	2e-146	Basidiomycota	n	Agaricomycetes	D
priority_effects_CL_48	-	-	0.06	12.00	-	0.14	0.16	-	3.09	*Coprinopsis marcescibilis*	DQ389728	1e-143	Basidiomycota	n	Agaricomycetes	D
priority_effects_CL_58	-	0.06	8.89	0.42	-	-	0.08	-	2.36	uncultured Agaricaceae	AM076653	6e-141	Basidiomycota	n	Agaricomycetes	D
priority_effects_CL_5	-	-	0.06	-	0.22	-	0.08	8.81	2.29	*Stereum hirsutum*	UDB017896	7E-155	Basidiomycota	n	n	D
priority_effects_CL_41	6.02	-	-	0.06	-	0.14	0.16	-	1.59	*Parasola conopilus*	UDB011851	4E-152	Basidiomycota	n	Agaricomycetes	D
priority_effects_CL_18	0.43	0.13	4.62	0.48	1.24	-	-	-	1.38	*Tubaria furfuracea*	UDB011741	2E-156	Basidiomycota	n	Agaricomycetes	D
priority_effects_CL_200	-	-	1.21	-	-	-	-	-	1.21	*Psathyrella prona*	FN396141	2e-145	Basidiomycota	n	Agaricomycetes	D
priority_effects_CL_1903	-	-	-	-	-	-	-	0.88	0.88	*Hypholoma fasciculare*	JQ685719	1e-58	Basidiomycota	n	Agaricomycetes	D
priority_effects_CL_23	1.70	0.06	2.37	0.06	-	-	0.08	-	0.85	uncultured Basidiomycete	KF297213	2e-94	Basidiomycota	n	Agaricomycetes	D
priority_effects_CL_136	-	-	-	-	-	-	0.62	-	0.62	*Lecythophora sp olrim15*	AY789083	2e-124	Basidiomycota	n	Dacrymycetes	D
priority_effects_CL_44	1.95	-	0.06	0.18	-	-	0.16	-	0.59	*Phlebia uda*	AF141614	7e-150	Basidiomycota	n	Agaricomycetes	D
priority_effects_CL_53	1.46	-	-	0.06	0.11	-	-	-	0.54	*Mycena sp MCVE997*	JF908495	8e-160	Basidiomycota	n	Agaricomycetes	D
priority_effects_CL_65	-	-	0.35	-	-	-	-	-	0.35	*Parasola schroeterii*	JN943136	2E-146	Basidiomycota	n	Agaricomycetes	D
priority_effects_CL_185	-	-	-	-	-	-	0.39	0.22	0.30	*Calocera cornea*	AY789083	2e-114	Basidiomycota	n	Dacrymycetes	D
priority_effects_CL_6	0.06	0.50	-	0.06	0.34	0.56	-	0.22	0.29	*Bjerkandera adusta*	JX082339	2e-146	Basidiomycota	n	Agaricomycetes	D
priority_effects_CL_273	-	-	-	-	-	0.28	-	-	0.28	*Massarina sp JP2013*	JX981477	5e-126	Basidiomycota	n	Tremellomycetes	D
priority_effects_CL_324	-	-	-	-	-	0.28	-	-	0.28	*Tremella foliacea*	UDB018809	6E-130	Basidiomycota	n	Agaricomycetes	D
priority_effects_CL_94	-	-	-	-	0.11	0.42	-	-	0.27	*Dacrymyces minor*	AB712458	9e-54	Basidiomycota	n	Dacrymycetes	D
priority_effects_CL_124	0.24	-	-	-	-	-	-	-	0.24	*Sistotrema sp quinchao*	KC514823	8e-110	Basidiomycota	n	n	D
priority_effects_CL_341	0.24	-	-	-	-	-	-	-	0.24	*Coprinellus callinus*	JN159518	1e-153	Basidiomycota	n	Agaricomycetes	D
priority_effects_CL_2601	0.24	-	-	-	-	-	-	-	0.24	unknown Agaricomycete	UDB018605	4e-127	Basidiomycota	n	Agaricomycetes	D
priority_effects_CL_144	-	-	-	0.24	-	-	-	-	0.24	*Psathyrella microrrhiza*	FN396130	7e-155	Basidiomycota	n	Agaricomycetes	D
priority_effects_CL_10	0.12	0.13	-	0.06	-	0.14	0.54	0.44	0.24	*Trametes versicolor*	UDB011614	3E-149	Basidiomycota	n	Agaricomycetes	D
priority_effects_CL_1881	-	-	-	-	0.22	-	-	-	0.22	uncultured Polyporales	FJ820656	5e-69	Basidiomycota	n	n	D
priority_effects_CL_59	0.49	-	0.17	0.12	-	-	0.08	-	0.21	uncultured Basidiomycete	AB370328	5e-107	Basidiomycota	n	n	D
priority_effects_CL_99	0.18	-	-	-	-	-	-	-	0.18	uncultured Basidiomycete	DQ309168	6e-151	Basidiomycota	n	n	D
priority_effects_CL_259	-	-	-	-	-	0.14	-	0.22	0.18	*Dacrymyces minor*	AB712458	7e-45	Basidiomycota	n	Dacrymycetes	D
priority_effects_CL_123	-	0.06	-	-	-	0.28	-	-	0.17	uncultured Agaricomycete	HQ211619	3e-113	Basidiomycota	n	n	D
priority_effects_CL_190	-	0.06	-	-	-	0.28	-	-	0.17	*Dacrymyces minor*	AB712458	9e-49	Basidiomycota	n	Dacrymycetes	D
priority_effects_CL_64	0.06	0.32	-	-	0.11	0.14	-	0.22	0.17	*Trametes palisotii*	EU661873	3e-59	Basidiomycota	n	Agaricomycetes	D
priority_effects_CL_181	-	-	-	-	-	-	0.16	-	0.16	Stephanosporaceae	UDB014037	4e-133	Basidiomycota	n	Tremellomycetes	D
priority_effects_CL_103	0.18	-	0.12	-	-	-	-	-	0.15	unknown Basidiomycete	KF673759	2e-125	Basidiomycota	n	Agaricomycetes	D
priority_effects_CL_110	-	-	-	-	0.22	0.14	0.08	-	0.15	*Dacrymyces subarcticus*	AB712467	4e-77	Basidiomycota	n	Dacrymycetes	D
priority_effects_CL_156	-	0.13	-	-	-	-	-	-	0.13	uncultured Basidiomycete	DQ309168	1e-142	Basidiomycota	n	n	D
priority_effects_CL_86	0.12	-	-	-	-	-	-	-	0.12	uncultured Basidiomycete	AB370321	5e-112	Basidiomycota	n	n	D
priority_effects_CL_143	0.12	-	-	-	-	-	-	-	0.12	uncultured Basidiomycete	KF385280	4E-132	Basidiomycota	n	Agaricomycetes	D
priority_effects_CL_77	-	-	0.12	-	-	-	-	-	0.12	uncultured Basidiomycete	HQ154321	2e-150	Basidiomycota	n	Agaricomycetes	D
priority_effects_CL_2419	-	-	0.12	-	-	-	-	-	0.12	unknown Agaricomycete	UDB018605	1e-98	Basidiomycota	n	Agaricomycetes	D
priority_effects_CL_22	-	-	0.06	0.06	0.11	-	-	0.22	0.11	*Vuilleminia comedens*	HM046892	3E-148	Basidiomycota	n	Agaricomycetes	D
priority_effects_CL_21	-	-	-	-	0.11	-	-	-	0.11	*Hypholoma fasciculare*	UDB011502	3E-159	Basidiomycota	n	Agaricomycetes	D
priority_effects_CL_385	-	-	-	-	-	0.14	0.08	-	0.11	Basidiomycota spDi2824	KC514899	5e-37	Basidiomycota	n	n	D

OTU ID no.	Occurrence as % of total sequences occurring in each precoloniser species								Average occurrence	Identification details			Taxonomy			Functional group
	Control	*V.comedens*	*H. fragiforme*	*B. nummularia*	*T. versicolor*	*S. hirsutum*	*B. adusta*	*H. fasciculare*		Taxon name	Accession #	E-value	Phylum	Subphylum	Class	
priority_effects_CL_130	-	-	0.12	-	-	-	0.08	-	0.10	uncultured Basidiomycete	EU517045	1e-106	Basidiomycota	n	n	D
priority_effects_CL_365	-	-	0.12	0.06	-	-	-	-	0.09	uncultured fungus	KF297177	1e-37	Basidiomycota	n	Agaricomycetes	D
priority_effects_CL_0	26.64	1.20	18.82	21.03	2.70	0.84	14.93	1.32	10.94	*Lasiosphaeris hispida*	JN942173	1e-126	Ascomycota	Pezizomycotina	Sordariomycetes	C
priority_effects_CL_1	1.09	2.90	5.02	3.15	26.10	26.97	10.96	7.71	10.49	*Phialocephala dimorphospora*	AB671498	1E-122	Ascomycota	Pezizomycotina	Leotiomycetes	C
priority_effects_CL_2	9.85	0.44	13.51	15.21	-	0.98	0.70	0.44	5.88	uncultured Ascomycete	JF449882	1e-122	Ascomycota	Pezizomycotina	Leotiomycetes	C
priority_effects_CL_8	0.30	0.38	0.35	-	7.20	5.76	18.82	5.07	5.41	*Chaetosphaeria innumera*	AF178551	1e-116	Ascomycota	Pezizomycotina	Sordariomycetes	C
priority_effects_CL_19	-	2.65	-	-	0.11	15.73	0.23	-	4.68	*Hyphodiscus hymeniophilus*	GU727550	5e-126	Ascomycota	Pezizomycotina	Leotiomycetes	C
priority_effects_CL_165	-	-	-	-	-	4.49	-	-	4.49	*Hyphodiscus hymeniophilus*	GU727555	5e-121	Ascomycota	Pezizomycotina	Leotiomycetes	C
priority_effects_CL_35	4.20	-	-	-	-	-	-	-	4.20	uncultured fungus	JX915518	8e-109	Ascomycota	n	n	C
priority_effects_CL_3	0.24	18.41	0.06	-	-	0.14	0.54	-	3.88	*Helotiales sp SGSGf25*	EU715657	8e-124	Ascomycota	Pezizomycotina	Leotiomycetes	C
priority_effects_CL_68	-	-	-	-	0.11	-	-	5.95	3.03	Helotiales sp JP2013	JX981510	2e-125	Ascomycota	Pezizomycotina	Dothideomycetes	C
priority_effects_CL_125	-	-	-	-	2.92	-	-	-	2.92	*Menispora ciliata*	EU488737	1E-097	Ascomycota	Pezizomycotina	Sordariomycetes	C
priority_effects_CL_7	0.12	3.47	10.10	1.09	0.56	0.14	3.81	1.10	2.55	*Chalara sp mh10666*	GQ996180	8e-124	Ascomycota	n	n	C
priority_effects_CL_17	0.06	11.16	1.21	-	1.24	0.42	2.33	0.66	2.44	*Hypocrea pachybasioides*	KC884809	7e-135	Ascomycota	n	n	C
priority_effects_CL_73	-	-	-	2.12	-	-	-	-	2.12	uncultured Ascomycete	KC588589	8e-124	Ascomycota	n	n	C
priority_effects_CL_14	0.12	-	0.35	5.64	-	-	-	-	2.03	*Lasiosphaeria lanuginosa*	AY587918	8e-124	Ascomycota	Pezizomycotina	Sordariomycetes	C
priority_effects_CL_25	0.61	0.44	0.69	0.24	1.24	6.88	3.97	1.32	1.92	*Lecythophora sp olrim15*	AY781228	5e-106	Ascomycota	Pezizomycotina	Sordariomycetes	C
priority_effects_CL_11	2.07	3.85	0.06	0.67	2.02	-	2.33	-	1.83	*Meliniomyces sp mh34632*	GQ996162	8e-124	Ascomycota	n	n	C
priority_effects_CL_12	0.12	-	-	0.06	6.41	0.14	2.26	-	1.80	uncultured Ascomycete	HM030597	6e-115	Ascomycota	n	n	C
priority_effects_CL_51	-	0.06	-	-	4.84	0.56	1.79	0.88	1.63	*Chaetosphaeria chloroconia*	AF178542	2e-124	Ascomycota	Pezizomycotina	Sordariomycetes	C
priority_effects_CL_132	-	-	-	-	1.69	1.26	-	-	1.48	*Cryptodiscus microstomus*	FJ904676	2e-124	Ascomycota	Pezizomycotina	Lecanoromycetes	C
priority_effects_CL_102	-	-	-	-	2.59	0.28	-	-	1.43	*Zignoella pulviscula*	AF178543	5e-106	Ascomycota	Pezizomycotina	Sordariomycetes	C
priority_effects_CL_16	0.06	0.13	0.06	-	5.29	-	1.48	-	1.40	uncultured Ascomycete	JX860449	2e-119	Ascomycota	Pezizomycotina	Leotiomycetes	C
priority_effects_CL_78	-	-	-	-	-	-	-	1.10	1.10	uncultured Ascomycete	JQ313083	1e-112	Ascomycota	Pezizomycotina	Leotiomycetes	C
priority_effects_CL_71	-	-	-	-	1.80	-	0.39	-	1.09	uncultured Ascomycete	AY699673	3e-88	Ascomycota	n	n	C
priority_effects_CL_119	-	-	1.04	-	-	-	-	-	1.04	uncultured Ascomycete	JX860440	1e-116	Ascomycota	Pezizomycotina	Sordariomycetes	C
priority_effects_CL_263	-	-	-	-	-	0.98	-	-	0.98	*Cryptodiscus microstomus*	FJ904676	5e-111	Ascomycota	Pezizomycotina	Lecanoromycetes	C
priority_effects_CL_55	-	-	0.06	-	1.12	0.28	2.95	0.22	0.93	uncultured Ascomycete	HQ212094	1e-77	Ascomycota	Pezizomycotina	Leotiomycetes	C
priority_effects_CL_36	-	1.58	-	-	2.14	0.28	0.39	0.22	0.92	*Haplographium catenatum*	FJ839621	1e-121	Ascomycota	Pezizomycotina	Leotiomycetes	C
priority_effects_CL_116	-	-	-	-	-	-	-	0.88	0.88	*Cryptococcus sp ATCCMYA4669*	KC171331	3e-78	Ascomycota	Pezizomycotina	Leotiomycetes	C
priority_effects_CL_24	1.70	-	1.62	0.06	-	0.14	-	-	0.88	uncultured Ascomycete	HQ433062	2e-104	Ascomycota	Pezizomycotina	Leotiomycetes	C
priority_effects_CL_37	1.09	-	0.52	-	-	-	-	-	0.81	uncultured Helotiales	JF449728	1e-96	Ascomycota	Pezizomycotina	Leotiomycetes	C
priority_effects_CL_33	0.06	0.32	-	-	1.01	-	0.86	1.76	0.80	*Haplographium catenatum*	FJ839622	2e-119	Ascomycota	Pezizomycotina	Leotiomycetes	C
priority_effects_CL_29	0.30	0.06	0.75	2.06	-	-	-	-	0.79	*Cylindrotrichum hennebertii*	AF178560	2e-115	Ascomycota	Pezizomycotina	Sordariomycetes	C
priority_effects_CL_20	1.82	-	0.35	-	-	-	0.08	-	0.75	*Cadophora malorum*	DQ404350	8E-124	Ascomycota	Pezizomycotina	Leotiomycetes	C
priority_effects_CL_128	-	-	-	-	-	1.26	-	0.22	0.74	*Lecythophora sp olrim15*	AY781228	2e-89	Ascomycota	Pezizomycotina	Sordariomycetes	C
priority_effects_CL_88	2.31	0.06	0.35	-	-	0.14	-	-	0.72	*uncultured Ascomycete*	HQ433062	3e-103	Ascomycota	n	n	C
priority_effects_CL_97	-	-	-	-	-	-	-	0.66	0.66	uncultured Ascomycete	HQ873357	4e-102	Ascomycota	n	n	C
priority_effects_CL_2218	-	-	-	-	-	-	-	0.66	0.66	*Lecythophora sp olrim15*	AY781228	5e-111	Ascomycota	Pezizomycotina	Sordariomycetes	C
priority_effects_CL_167	-	-	-	-	0.90	0.42	-	-	0.66	uncultured Ascomycete	DQ914715	2e-49	Ascomycota	Pezizomycotina	Leotiomycetes	C
priority_effects_CL_66	-	0.13	-	-	0.11	1.69	-	0.66	0.65	uncultured Ascomycete	KC965986	1e-112	Ascomycota	n	n	C
priority_effects_CL_46	-	1.20	-	-	0.11	0.70	0.39	-	0.60	*Lecythophora sp olrim15*	AY781228	5e-96	Ascomycota	Pezizomycotina	Sordariomycetes	C
priority_effects_CL_316	-	-	0.58	-	-	-	-	-	0.58	uncultured Ascomycete	AM260896	5e-106	Ascomycota	Pezizomycotina	Leotiomycetes	C
priority_effects_CL_32	0.91	-	0.12	0.55	-	-	-	-	0.52	*Sordariales sp 7SAp341*	AB746927	3e-123	Ascomycota	Pezizomycotina	Sordariomycetes	C
priority_effects_CL_104	-	0.06	-	-	1.35	0.14	-	-	0.52	*Phialophora clavispora*	AB190868	2e-125	Ascomycota	Pezizomycotina	Eurotiomycetes	C
priority_effects_CL_60	0.55	1.32	0.06	-	-	0.14	-	-	0.52	uncultured Ascomycete	GU309234	4e-122	Ascomycota	n	n	C
priority_effects_CL_79	0.49	-	-	-	-	-	-	-	0.49	*Ascocoryne sarcoides*	HM152550	8e-124	Ascomycota	Pezizomycotina	Leotiomycetes	C
priority_effects_CL_54	-	1.13	0.17	-	0.11	-	-	-	0.47	uncultured Ascomycete	GQ996132	2e-114	Ascomycota	n	n	C
priority_effects_CL_27	0.79	0.38	0.64	0.48	-	-	0.08	-	0.47	uncultured Ascomycete	JX535087	5e-116	Ascomycota	n	n	C
priority_effects_CL_69	0.06	-	0.87	-	-	-	-	-	0.46	*Haptocillium sinense*	AJ292417	2e-105	Ascomycota	Pezizomycotina	Sordariomycetes	C

OTU ID no.	Occurrence as % of total sequences occurring in each precoloniser species								Average occurrence	Identification details			Taxonomy			Functional group
	Control	*V.comedens*	*H. fragiforme*	*B. nummularia*	*T. versicolor*	*S. hirsutum*	*B. adusta*	*H. fasciculare*		Taxon name	Accession #	E-value	Phylum	Subphylum	Class	
priority_effects_CL_174	0.06	-	0.87	-	-	-	-	-	0.46	uncultured Ascomycete	DQ273345	3e-108	Ascomycota	Pezizomycotina	Sordariomycetes	C
priority_effects_CL_62	-	0.44	-	-	-	-	-	-	0.44	uncultured Ascomycete	FJ265919	5e-17	Ascomycota	n	n	C
priority_effects_CL_152	-	-	-	-	-	-	-	0.44	0.44	uncultured fungus	JQ313083	1e-101	Ascomycota	Pezizomycotina	Leotiomycetes	C
priority_effects_CL_177	-	-	-	-	-	-	-	0.44	0.44	uncultured Ascomycete	FJ820734	1e-132	Ascomycota	n	n	C
priority_effects_CL_28	-	-	-	-	-	0.14	0.08	1.10	0.44	*Hypoxylon fragiforme*	JN979420	2e-134	Ascomycota	Pezizomycotina	Sordariomycetes	C
priority_effects_CL_42	1.16	-	0.29	0.12	-	0.14	-	-	0.43	*Calosphaeriales sp ICMP17422*	EU770223	5e-111	Ascomycota	Pezizomycotina	Sordariomycetes	C
priority_effects_CL_30	0.06	0.32	0.40	0.06	0.56	0.98	0.78	0.22	0.42	*Lecythophora sp OTU030*	HE998721	2e-110	Ascomycota	Pezizomycotina	Sordariomycetes	C
priority_effects_CL_105	-	-	-	-	-	0.42	-	-	0.42	*Lecythophora sp olrim15*	AY781228	8e-94	Ascomycota	Pezizomycotina	Sordariomycetes	C
priority_effects_CL_43	0.06	-	0.35	0.85	-	-	-	-	0.42	*Massarina rubi*	AF383963	2E-130	Ascomycota	Pezizomycotina	Dothideomycetes	C
priority_effects_CL_50	1.03	-	0.12	0.06	-	-	-	-	0.40	*Phaeoacremonium austroafricanum*	EU552158	2e-135	Ascomycota	Pezizomycotina	Sordariomycetes	C
priority_effects_CL_52	0.06	1.07	0.06	-	-	-	-	-	0.40	uncultured fungus	KC588551	4e-117	Ascomycota	n	n	C
priority_effects_CL_47	-	-	0.64	0.12	-	-	-	-	0.38	uncultured Ascomycete	GQ921793	8e-110	Ascomycota	n	n	C
priority_effects_CL_49	-	0.50	-	-	0.67	0.14	0.16	-	0.37	*Cladosporium sp CBS12588*	AF050264	2e-135	Ascomycota	Pezizomycotina	Dothideomycetes	C
priority_effects_CL_92	-	-	0.12	0.67	0.22	-	-	-	0.34	uncultured Ascomycete	EF218791	6e-95	Ascomycota	Pezizomycotina	Leotiomycetes	C
priority_effects_CL_56	0.79	-	0.06	0.12	-	-	-	-	0.32	uncultured Ascomycete	KC588635	3e-108	Ascomycota	n	n	C
priority_effects_CL_87	-	-	-	-	0.11	0.14	0.70	-	0.32	*Lecanoromycetes sp CHAa18*	EF373561	4e-87	Ascomycota	n	n	C
priority_effects_CL_115	-	0.32	-	-	-	-	-	-	0.32	*Dactylella rhopalota*	DQ494370	2E-139	Ascomycota	Pezizomycotina	Orbiliomycetes	C
priority_effects_CL_127	-	0.32	-	-	-	-	-	-	0.32	uncultured Ascomycete	HQ432991	2e-100	Ascomycota	n	n	C
priority_effects_CL_61	0.18	0.32	0.58	0.18	-	-	-	-	0.31	uncultured Ascomycete	KC588645	4e-127	Ascomycota	n	n	C
priority_effects_CL_111	-	0.57	0.06	-	-	-	-	-	0.31	uncultured Ascomycete	KC588586	8e-129	Ascomycota	n	n	C
priority_effects_CL_98	0.30	-	-	-	-	-	-	-	0.30	*Neonectria hubeiensis*	FJ560439	2e-130	Ascomycota	Pezizomycotina	Sordariomycetes	C
priority_effects_CL_108	0.30	-	-	-	-	-	-	-	0.30	uncultured Ascomycete	KC588574	8e-119	Ascomycota	n	n	C
priority_effects_CL_63	-	-	0.29	-	-	-	-	-	0.29	*Cadophora fastigiata*	KC514850	2e-125	Ascomycota	n	n	C
priority_effects_CL_135	0.18	0.25	0.40	0.18	0.45	0.42	0.08	-	0.28	*Exophiala sp TU181006*	AY781228	5e-116	Ascomycota	Pezizomycotina	Sordariomycetes	C
priority_effects_CL_147	-	-	-	-	0.11	-	-	0.44	0.28	uncultured Ascomycete	HQ433088	2e-134	Ascomycota	n	n	C
priority_effects_CL_101	0.06	0.06	-	-	0.22	0.70	-	-	0.26	*Pyrenochaeta sp AU/DBT237*	KC963916	8e-129	Ascomycota	n	n	C
priority_effects_CL_109	-	0.25	-	-	-	-	-	-	0.25	*Pithomyces valparadisiacus*	EU552152	9e-69	Ascomycota	Pezizomycotina	Dothideomycetes	C
priority_effects_CL_40	0.06	-	0.64	0.06	-	-	-	-	0.25	uncultured Ascomycete	JF449592	3e-123	Ascomycota	Pezizomycotina	Sordariomycetes	C
priority_effects_CL_95	-	0.44	-	0.06	-	-	-	-	0.25	uncultured Ascomycete	JQ346840	4e-102	Ascomycota	Pezizomycotina	Leotiomycetes	C
priority_effects_CL_75	-	0.38	0.12	-	-	-	-	-	0.25	uncultured Ascomycete	DQ447998	1e-116	Ascomycota	n	n	C
priority_effects_CL_83	0.24	-	-	-	-	-	-	-	0.24	uncultured Ascomycete	JF449878	2e-130	Ascomycota	Pezizomycotina	Sordariomycetes	C
priority_effects_CL_80	0.24	-	0.40	0.06	-	-	-	-	0.24	*Arbusculina fragmentans*	KC834042	4e-107	Ascomycota	n	n	C
priority_effects_CL_70	-	-	0.23	-	-	-	-	-	0.23	*Arbusculina fragmentans*	KC834042	4e-112	Ascomycota	n	n	C
priority_effects_CL_96	-	0.38	-	-	-	-	0.08	-	0.23	uncultured Ascomycete	KC588610	2e-95	Ascomycota	n	n	C
priority_effects_CL_107	-	-	-	-	0.22	-	-	-	0.22	uncultured Ascomycete	KC965795	1e-66	Ascomycota	n	n	C
priority_effects_CL_397	-	-	-	-	0.22	-	-	-	0.22	*Cryptodiscus microstomus*	FJ904676	5e-121	Ascomycota	Pezizomycotina	Lecanoromycetes	C
priority_effects_CL_45	-	0.19	0.06	-	-	-	0.16	0.44	0.21	uncultured Ascomycete	JX860474	2e-125	Ascomycota	Pezizomycotina	Leotiomycetes	C
priority_effects_CL_91	-	-	0.35	0.06	-	-	-	-	0.20	uncultured Ascomycete	GQ921793	4e-112	Ascomycota	n	n	C
priority_effects_CL_67	0.24	-	0.29	0.06	-	-	-	-	0.20	*Coniochaetaceae sp RS050*	EU082785	9e-64	Ascomycota	Pezizomycotina	Sordariomycetes	C
priority_effects_CL_229	-	0.19	-	-	-	-	-	-	0.19	uncultured Ascomycete	GU309234	5e-111	Ascomycota	n	n	C
priority_effects_CL_265	-	0.19	-	-	-	-	-	-	0.19	uncultured Ascomycete	JQ312710	1e-117	Ascomycota	n	n	C
priority_effects_CL_121	0.18	-	-	-	-	-	-	-	0.18	uncultured Ascomycete	KC588576	2e-125	Ascomycota	n	n	C
priority_effects_CL_129	0.18	-	-	-	-	-	-	-	0.18	uncultured Ascomycete	KC588649	3e-138	Ascomycota	Pezizomycotina	Sordariomycetes	C
priority_effects_CL_149	-	-	-	0.18	-	-	-	-	0.18	uncultured Ascomycete	JF433007	5e-121	Ascomycota	Pezizomycotina	Sordariomycetes	C
priority_effects_CL_233	-	-	-	0.18	-	-	-	-	0.18	uncultured Ascomycete	GQ996173	8e-104	Ascomycota	n	n	C
priority_effects_CL_74	0.24	-	0.12	0.18	-	-	-	-	0.18	*Phaeoacremonium rubrigenum*	AJ575811	5e-131	Ascomycota	Pezizomycotina	Sordariomycetes	C
priority_effects_CL_90	0.12	-	0.35	0.06	-	-	-	-	0.18	*Cosmospora sp NRRL28291*	JN615481	4e-132	Ascomycota	Pezizomycotina	Sordariomycetes	C
priority_effects_CL_141	-	-	0.17	-	-	-	-	-	0.17	uncultured Ascomycete	JF449882	2e-109	Ascomycota	Pezizomycotina	Leotiomycetes	C
priority_effects_CL_223	-	-	-	-	0.11	-	-	0.22	0.17	*Haplographium catenatum*	FJ839622	5e-101	Ascomycota	Pezizomycotina	Leotiomycetes	C
priority_effects_CL_131	-	-	-	0.18	-	0.14	-	-	0.16	*Chaetosphaeria tulasneorum*	AF178547	6e-125	Ascomycota	Pezizomycotina	Sordariomycetes	C

OTU ID no.	Occurrence as % of total sequences occurring in each precoloniser species								Average occurrence	Identification details			Taxonomy			Functional group
	Control	*V.comedens*	*H. fragiforme*	*B. nummularia*	*T. versicolor*	*S. hirsutum*	*B. adusta*	*H. fasciculare*		Taxon name	Accession #	E-value	Phylum	Subphylum	Class	
priority_effects_CL_330	-	-	-	-	0.11	0.14	-	0.22	0.16	uncultured Ascomycete	AB089668	2e-59	Ascomycota	n	n	C
priority_effects_CL_138	-	-	-	-	-	-	0.16	-	0.16	*Cristinia helvetica*	UDB016420	8e-130	Ascomycota	Pezizomycotina	Sordariomycetes	C
priority_effects_CL_150	-	-	-	-	-	-	0.16	-	0.16	*Chaetosphaeria sp 235OA2013*	JX535168	6e-125	Ascomycota	Pezizomycotina	Sordariomycetes	C
priority_effects_CL_314	-	-	-	-	-	-	0.16	-	0.16	*Chloridium lignicola*	AF178544	1e-117	Ascomycota	Pezizomycotina	Sordariomycetes	C
priority_effects_CL_702	-	-	-	-	-	-	0.16	-	0.16	*Haplographium catenatum*	FJ839622	3e-83	Ascomycota	Pezizomycotina	Leotiomycetes	C
priority_effects_CL_1842	-	-	-	-	-	-	0.16	-	0.16	*Pleosporales sp E02*	HQ115654	1e-126	Ascomycota	Pezizomycotina	Dothideomycetes	C
priority_effects_CL_57	0.24	-	-	0.06	-	-	-	-	0.15	*Pezizomycetes sp WF148*	HQ130704	2e-85	Ascomycota	Pezizomycotina	Pezizomycetes	C
priority_effects_CL_198	-	-	-	-	-	-	0.08	0.22	0.15	*Chloridium lignicola*	AF178544	1e-97	Ascomycota	Pezizomycotina	Sordariomycetes	C
priority_effects_CL_133	0.06	-	-	-	0.22	-	-	-	0.14	uncultured Ascomycete	HM044632	4e-102	Ascomycota	n	n	C
priority_effects_CL_158	-	0.06	-	-	-	0.14	-	0.22	0.14	uncultured Ascomycete	HQ433088	4e-122	Ascomycota	n	n	C
priority_effects_CL_222	0.12	-	-	-	-	0.14	-	-	0.13	uncultured Ascomycete	GU327446	1e-132	Ascomycota	Pezizomycotina	Sordariomycetes	C
priority_effects_CL_295	-	-	-	-	0.11	0.14	-	-	0.13	uncultured Ascomycete	EF521221	1e-116	Ascomycota	Pezizomycotina	Leotiomycetes	C
priority_effects_CL_100	-	0.13	-	-	-	-	-	-	0.13	*Menispora tortuosa*	AF178558	8e-114	Ascomycota	Pezizomycotina	Sordariomycetes	C
priority_effects_CL_142	-	0.13	-	-	-	-	-	-	0.13	uncultured Ascomycete	KC588652	2e-125	Ascomycota	Pezizomycotina	Sordariomycetes	C
priority_effects_CL_146	-	0.13	-	-	-	-	-	-	0.13	*Scytalidium lignicola*	GU934579	1E-122	Ascomycota	Pezizomycotina	Leotiomycetes	C
priority_effects_CL_155	-	0.13	-	-	-	-	-	-	0.13	*Chalara piceae-abietis*	FR667231	1e-101	Ascomycota	Pezizomycotina	Leotiomycetes	C
priority_effects_CL_247	-	0.13	-	-	-	-	-	-	0.13	*Dactylella sp AS60288*	DQ494363	7e-140	Ascomycota	Pezizomycotina	Orbiliomycetes	C
priority_effects_CL_275	-	0.13	-	-	-	-	-	-	0.13	unknown fungus	GQ996162	2e-105	Ascomycota	Pezizomycotina	Leotiomycetes	C
priority_effects_CL_1235	-	0.13	-	-	-	-	-	-	0.13	uncultured Ascomycete	KF297027	4e-132	Ascomycota	Pezizomycotina	Eurotiomycetes	C
priority_effects_CL_93	0.06	0.25	-	0.06	-	-	-	-	0.12	*Dactylella mammillata*	AY902794	4e-137	Ascomycota	Pezizomycotina	Orbiliomycetes	C
priority_effects_CL_81	0.12	-	-	-	-	-	-	-	0.12	uncultured Ascomycete	HM230871	4e-47	Ascomycota	Pezizomycotina	Leotiomycetes	C
priority_effects_CL_84	0.12	-	-	-	-	-	-	-	0.12	uncultured Ascomycete	KF296725	3e-118	Ascomycota	n	n	C
priority_effects_CL_117	0.12	-	-	-	-	-	-	-	0.12	uncultured Ascomycete	HQ433062	5e-96	Ascomycota	Pezizomycotina	Leotiomycetes	C
priority_effects_CL_134	0.06	-	-	0.18	-	-	-	-	0.12	*Exophiala sp*	EF115305	1e-147	Ascomycota	Pezizomycotina	Eurotiomycetes	C
priority_effects_CL_243	-	-	-	0.12	-	-	-	-	0.12	*Cylindrotrichum hennebertii*	AF178560	5e-106	Ascomycota	Pezizomycotina	Sordariomycetes	C
priority_effects_CL_411	-	-	-	0.12	-	-	-	-	0.12	*Massarina sp MUT4323*	KC339223	1e-107	Ascomycota	Pezizomycotina	Dothideomycetes	C
priority_effects_CL_520	-	-	-	0.12	-	-	-	-	0.12	*Phialocephala fusca*	AB671500	4E-082	Ascomycota	Pezizomycotina	Sordariomycetes	C
priority_effects_CL_82	-	0.13	-	-	0.11	-	-	-	0.12	*Hypocreales sp TR173*	HQ608153	2e-119	Ascomycota	Pezizomycotina	Sordariomycetes	C
priority_effects_CL_2040	-	-	0.12	-	-	-	-	-	0.12	*Trichoderma hamatum*	X93975	7e-130	Ascomycota	n	n	C
priority_effects_CL_2288	-	-	0.12	-	-	-	-	-	0.12	uncultured Ascomycete	HQ433062	8e-84	Ascomycota	Pezizomycotina	Leotiomycetes	C
priority_effects_CL_120	-	-	0.12	-	0.11	-	-	-	0.11	*Cladophialophora sp.*	DQ124131	9e-134	Ascomycota	n	n	C
priority_effects_CL_398	-	-	-	-	-	0.14	0.08	-	0.11	uncultured Ascomycete	KC978028	7e-110	Ascomycota	n	Dothideomycetes	C
priority_effects_CL_76	0.06	0.06	0.23	0.06	-	-	-	-	0.10	uncultured Ascomycete	JX915540	4e-122	Ascomycota	n	n	C
priority_effects_CL_85	-	0.19	0.06	0.06	-	-	-	-	0.10	*Calycina claroflava*	KC412006	1e-106	Ascomycota	Pezizomycotina	Leotiomycetes	C
priority_effects_CL_113	0.06	-	-	0.12	-	-	-	-	0.09	*Meliniomyces sp mh21992*	GQ996156	5e-121	Ascomycota	n	n	C
priority_effects_CL_170	0.06	-	0.06	-	0.11	-	-	-	0.08	unknown Boletales	NR103687	9e-134	Ascomycota	n	n	C
priority_effects_CL_189	0.06	-	-	-	-	-	0.08	-	0.07	uncultured Ascomycete	GU174433	1e-101	Ascomycota	n	n	C
priority_effects_CL_348	-	-	-	0.06	-	-	0.08	-	0.07	*Lasiosphaeris hispida*	JN942173	4e-87	Ascomycota	Pezizomycotina	Sordariomycetes	C
priority_effects_CL_183	0.06	0.06	-	-	-	-	-	-	0.06	*Helotiales sp 220OA2013*	JX535161	2e-119	Ascomycota	Pezizomycotina	Leotiomycetes	C
priority_effects_CL_322	0.06	0.06	-	-	-	-	-	-	0.06	*Lecythophora sp olrim15*	AY781228	1e-97	Ascomycota	Pezizomycotina	Sordariomycetes	C
priority_effects_CL_122	0.06	-	-	0.06	-	-	-	-	0.06	uncultured Ascomycete	EF635808	2e-126	Ascomycota	n	n	C
priority_effects_CL_2260	-	0.06	0.06	-	-	-	-	-	0.06	*Plenodomus influorescens*	JF740229	2e-125	Ascomycota	Pezizomycotina	Dothideomycetes	C
priority_effects_CL_126	0.06	-	0.06	0.06	-	-	-	-	0.06	uncultured Ascomycete	JF449882	5e-116	Ascomycota	Pezizomycotina	Leotiomycetes	C
priority_effects_CL_218	0.06	-	0.06	-	-	-	-	-	0.06	uncultured Ascomycete	JF519259	1e-121	Ascomycota	n	n	C
priority_effects_CL_220	0.06	-	0.06	-	-	-	-	-	0.06	uncultured Ascomycete	JF449649	2e-114	Ascomycota	Pezizomycotina	Leotiomycetes	C
priority_effects_CL_224	0.06	-	0.06	-	-	-	-	-	0.06	uncultured Ascomycete	KC753433	3e-123	Ascomycota	Pezizomycotina	Dothideomycetes	C
priority_effects_CL_212	0.06	-	-	-	0.11	0.00	-	-	0.06	uncultured Ascomycete	JN890098	9e-139	Ascomycota	n	n	C
priority_effects_CL_191	-	-	-	-	1.46	-	-	-	1.46	unknown fungus	KF212335	1e-126	n	n	n	
priority_effects_CL_26	-	3.97	-	0.06	1.01	-	1.56	0.22	1.36	uncultured fungus	KC588610	8e-114	n	n	n	
priority_effects_CL_173	-	-	-	-	-	1.26	-	-	1.26	uncultured fungus	KC588610	2e-89	n	n	n	

OTU ID no.	Occurrence as % of total sequences occurring in each precoloniser species								Average occurrence	Identification details			Taxonomy			Functional group
	Control	*V.comedens*	*H. fragiforme*	*B. nummularia*	*T. versicolor*	*S. hirsutum*	*B. adusta*	*H. fasciculare*		Taxon name	Accession #	E-value	Phylum	Subphylum	Class	
priority_effects_CL_31	-	0.19	-	0.06	0.45	5.20	0.23	0.22	1.06	uncultured fungus	KC588610	1e-97	n	n	n	
priority_effects_CL_38	1.52	-	-	-	0.11	0.14	-	-	0.59	uncultured fungus	KC588633	1e-102	n	n	n	
priority_effects_CL_89	0.43	0.32	-	-	-	-	0.08	0.88	0.42	uncultured fungus	AY704761	2e-100	n	n	n	
priority_effects_CL_347	-	-	0.23	-	-	-	-	-	0.23	uncultured fungus	AJ875348	1e-12	n	n	n	
priority_effects_CL_2606	-	-	-	-	-	-	0.16	-	0.16	unknown fungus	KF212281	1e-72	n	n	n	
priority_effects_CL_72	-	0.06	0.06	-	0.34	-	-	-	0.15	uncultured ectomycorrhizal	JX042982	5e-136	n	n	n	
priority_effects_CL_118	-	0.13	-	-	-	-	-	-	0.13	unknown	-	-	-	-	-	
priority_effects_CL_161	-	0.13	-	-	-	-	-	-	0.13	unknown	-	-	-	-	-	
priority_effects_CL_244	0.12	-	-	-	-	-	-	-	0.12	uncultured fungus	KC588633	2e-99	n	n	n	
priority_effects_CL_304	0.12	-	-	-	-	-	-	-	0.12	uncultured fungus	KC588633	2e-104	n	n	n	
