# Supplementary material for: Priority effects during fungal community establishment in beech wood
Source: ISME J. 2015 Mar 20;9(10):2246–60. doi: 10.1038/ismej.2015.38 (PMC4579477; doi:10.1038/ismej.2015.38)
Supplement: Supplementary Table 5 [file ismej201538x9.pdf]

Supplementary Table 5. Taxa recovered from different precoloniser species by reisolation and disk incubation. Each taxon is listed as a proportion of the total taxa recovered following each precoloniser species, and the overall occurrence of each taxon is shown. Named taxa were identified by ITS sequencing, and where pure culture or sequencing was not possible the taxa are described as groups of similar phenotype (G) or individuals (U), and their mycelial morphology described. The recovery method of each taxon refers to whether it was obtained through reisolation onto agar from wood (Reisol) or cords (Cords), or as outgrowth following disk incubation (Disk).

Taxon name/code	Occurrence		Occurrence as proportion of total cOTUs following each precoloniser species										Recovery method	Description	Accession number	Phylum
	No.	% total	Control	*V. comedens*	*H. fragiforme*	*B. nummularia*	*T. versicolor*	*S. hirsutum*	*B. adusta*	*H. fasciculare*	*P. velutina*	*P. impudicus*				
G 16	53	9.397	0.17	0.06	0.14	0.13	-	0.03	0.06	-	-	0.11	Disk	Matte black fluffy mycelium	-	-
Hypocrea avellanea	39	6.915	0.04	0.23	-	-	0.05	0.17	0.02	-	0.10	0.02	Reisol	Fluffy white mycelium, white balls in older regions	UDB011502	Basidiomycota
Phanerochaete	29	5.142	0.07	0.06	0.03	0.03	0.05	-	0.10	0.05	-	0.04	Reisol	Yellow/orange fluffy mycelium and cords	UDB017837	Basidiomycota
Xenasmatella vaga	23	4.078	0.05	0.01	0.03	0.06	-	0.10	0.08	-	-	0.07	Cords	Bright yellow mat of cords with white edges	UDB000519	Basidiomycota
G 35	19	3.369	0.03	0.03	0.05	-	0.14	-	-	-	0.10	-	Disk	Green bobbly mycelium with lots of spores	-	-
G 21	18	3.191	0.04	0.07	0.05	-	0.02	0.03	-	0.05	-	-	Disk	Orange spiny	-	-
G 23	17	3.014	-	0.05	0.03	0.04	0.05	-	0.06	-	-	0.02	Disk	Yellow/orange fluffy mycelium and cords	-	-
G 26	11	1.950	0.05	0.01	-	0.01	0.02	-	0.02	-	-	0.02	Disk	Yellow/orange fluffy mycelium with thick woolly cords	-	-
G 6	10	1.773	-	0.02	0.03	0.04	0.02	-	-	-	-	0.04	Disk	Fine cream/white mycelium	-	-
Parasola conopilus	10	1.773	0.02	0.04	-	0.01	0.02	0.03	-	-	-	0.02	Reisol	Butter yellow thick feathery cords	UDB011851	Basidiomycota
G 4	8	1.418	0.01	-	0.03	0.01	-	0.03	0.02	0.05	-	0.02	Disk	Dusty orange	-	-
Phlebia uda	8	1.418	0.07	-	-	-	-	-	-	-	-	-	Disk	Bright yellow mycelial fans	AB084621	Basidiomycota
Xenasmatella	8	1.418	-	0.02	0.02	0.01	0.04	0.07	-	-	-	-	Disk	Orange felt-like mycelium with spiny haphazard cords	UDB000519	Basidiomycota
G 13	7	1.241	-	0.01	-	0.01	0.04	0.03	0.02	-	-	0.02	Reisol	Bright pink/yellow fluffy mycelium	-	-
G 17	7	1.241	0.01	0.01	0.02	-	-	0.03	0.06	-	-	-	Disk	Mustard yellow mycelium and cords	-	-
G 33	7	1.241	0.06	-	-	-	-	-	-	-	-	-	Disk	Thin white mycelium	-	-
G 8	7	1.241	0.03	0.01	0.02	-	-	-	0.02	-	-	-	Disk	Fine white cords	-	-
Absidia glauca	6	1.064	-	-	-	-	-	-	0.10	0.05	-	-	Disk	Antler-like cream protuberances	-	Ascomycota
Ceratocystis paradoxa	6	1.064	-	0.01	-	0.01	-	-	0.02	-	-	0.07	Reisol	Slightly fluffy white mycelium with brown pig	KC305160	Ascomycota
G 1	6	1.064	-	0.02	0.02	0.01	-	-	-	0.10	-	-	Reisol	Brown/black fluffy fungus with white aerial	JQ809674	Ascomycota
G 24	6	1.064	0.01	0.01	-	-	0.02	-	0.02	-	0.10	0.02	Disk	Yellow/orange fluffy mycelium and cords, older regions pigmented dark orange	-	-
G 32	6	1.064	-	-	0.03	-	-	0.03	0.02	-	0.10	0.02	Disk	Thin glass-like cords	-	-
G 27	5	0.887	0.01	-	-	-	-	0.03	0.02	-	-	0.04	Disk	Tan thin haphazard cords	-	-
G 28	5	0.887	0.01	-	-	-	0.02	0.03	0.02	-	-	0.02	Disk	Thick orange cords	-	-
Hypholoma fasciculare	5	0.887	-	0.01	0.02	-	0.04	-	-	0.05	-	-	Reisol	Thick white cords	EU715657	Ascomycota
Mortierella	5	0.887	-	-	-	0.03	0.04	-	-	-	0.10	-	Reisol	Patchy white mycelium that seems to grow in waves	AY154690	Ascomycota
Mortierella parvispora	5	0.887	-	-	0.02	0.03	-	-	-	-	0.10	0.02	Reisol	Patchy white mycelium that seems to grow in waves	-	Ascomycota
Scopuloides hydnoides	5	0.887	-	-	0.03	-	0.02	-	-	-	-	0.04	Reisol	Feathery yellow cords	UDB016378	Basidiomycota
Fusarium lateritium	4	0.709	-	0.02	-	0.01	0.02	-	-	-	-	-	Reisol	Bright pink/yellow fluffy mycelium	JQ693397	Ascomycota
Fusarium sp	4	0.709	0.01	0.01	-	0.01	-	-	-	0.05	-	-	Reisol	Bright pink fluffy mycelium	HQ630964	Ascomycota
G 11	4	0.709	0.02	-	-	-	-	-	-	-	-	0.04	Disk	Flat white mycelium	-	-
G 3	4	0.709	-	0.01	-	0.01	-	0.03	0.02	-	-	-	Disk	Dense yellow/orange cords	-	-
G 34	4	0.709	-	0.01	-	-	0.02	-	-	0.10	-	-	Disk	Tough brown cords	-	-
G 37	4	0.709	0.01	-	0.03	0.01	-	-	-	-	-	-	Disk	White mycelium and spores	-	-
G 38	4	0.709	0.01	-	-	-	0.04	-	0.02	-	-	-	Disk	White spiny	-	-
G 5	4	0.709	-	0.01	-	-	-	0.03	0.02	-	0.10	-	Disk	Fine cream cords	-	-
G12	4	0.709	0.01	0.01	-	0.01	-	-	0.02	-	-	-	Disk	Fluffy white mycelium	-	-
uncultured Basidiomycota| *Clitopilus*	4	0.709	-	-	-	-	0.05	-	-	-	-	0.02	Reisol	Fine lace-like white mycelium	GU328568	Basidiomycota

Taxon name/code	Occurrence		Occurrence as proportion of total cOTUs following each precoloniser species										Recovery method	Description	Accession number	Phylum
	No.	% total	Control	*V. comedens*	*H. fragiforme*	*B. nummularia*	*T. versicolor*	*S. hirsutum*	*B. adusta*	*H. fasciculare*	*P. velutina*	*P. impudicus*				
Armillaria-type cords	3	0.532	0.01	-	-	-	-	0.03	-	-	-	0.02	Disk	Thick brown rubbery/woody cords	-	Basidiomycota
G 18	3	0.532	-	-	-	-	-	-	0.02	-	-	0.04	Disk	Orange bobbly mycelium	-	-
G 19	3	0.532	-	-	0.02	-	-	-	0.02	-	-	0.02	Disk	Orange cords with lighter edges	-	-
G 2	3	0.532	-	0.01	-	-	-	0.07	-	-	-	-	Disk	Clear cords like cobweb	-	-
G 20	3	0.532	0.02	-	-	-	-	-	-	-	-	0.02	Disk	Orange fluffy mycelium with thick cords	-	-
G 30	3	0.532	-	-	0.03	-	-	0.03	-	-	-	-	Disk	Thick yellow cords	-	-
G 36	3	0.532	0.01	0.01	0.02	-	-	-	-	-	-	-	Disk	Thick white rubbery mycelium	-	-
Helotiales sp SGSGf25	3	0.532	-	0.02	-	-	-	0.03	-	-	-	-	Reisol	White fluffy edges with yellow pigmentation i	KC581300	Basidiomycota
Mortierella sp 12NJ01	3	0.532	-	-	0.03	-	-	-	-	-	-	0.02	Reisol	Patchy white mycelium that seems to grow in waves	JX976049	Ascomycota
Mycena galopus	3	0.532	0.01	-	0.02	-	0.02	-	-	-	-	-	Reisol	Fine white mycelium and cords	HM240534	Basidiomycota
Psathyrella candolleana	3	0.532	0.03	-	-	-	-	-	-	-	-	-	Disk	Cream fluffy mycelium	EU520251	Basidiomycota
uncultured fungus|Leotiomycetes	3	0.532	-	-	0.02	-	0.02	-	0.02	-	-	-	Reisol	Grey/brown fluffy mycelium	-	Ascomycota
uncultured *Fusarium*	3	0.532	-	0.01	-	0.01	0.02	-	-	-	-	-	Reisol	Fluffy white mycelium with peach/orange pigmentation in centre	KF385354	Ascomycota
Agaricomycetes – *P. vaga*	2	0.355	-	-	-	-	-	-	0.04	-	-	-	Reisol	Cream fluffy mycelium and cords	UDB018605	Basidiomycota
Botryotinia sp FQ 4 1	2	0.355	-	-	-	0.01	-	-	-	0.05	-	-	Reisol	Green/grey mycelium surrounded by vivid yellow pigmentation	KF438015	Ascomycota
Diplodia mutila	2	0.355	-	-	0.02	0.01	-	-	-	-	-	-	Reisol	Black/grey pigmented with fluffy white aerial mycelium	KF225531	Ascomycota
Dothiorella iberica	2	0.355	-	-	0.02	0.01	-	-	-	-	-	-	Reisol	Green/grey with pigment in agar and birght white aerial mycelium	JQ411407	Ascomycota
Epicoccum sp 629 AI 2013	2	0.355	-	-	-	-	0.02	-	0.02	-	-	-	Reisol	Dark orange/red/brown, highly pigmented	KC662229	Ascomycota
fungal sp APA 2013	2	0.355	-	-	-	0.01	-	0.03	-	-	-	-	Reisol	Light pink fluffy mycelium	KF212335	Ascomycota
G 10	2	0.355	-	-	-	-	0.02	-	-	-	0.10	-	Disk	Flat grey mycelium	-	-
G 14	2	0.355	-	-	-	-	-	-	-	0.10	-	-	Reisol	Grey patchy mycelium	-	-
G 15	2	0.355	-	-	-	0.03	-	-	-	-	-	-	Disk	Matte yellow dense cords	-	-
G 22	2	0.355	-	0.01	-	-	0.02	-	-	-	-	-	Disk	Peach spiny cords	-	-
G 25	2	0.355	0.01	0.01	-	-	-	-	-	-	-	-	Disk	Yellow/orange fluffy mycelium with thin white cords	-	-
G 29	2	0.355	-	-	-	-	-	-	-	0.10	-	-	Disk	Yellow leathery cords	-	-
G 31	2	0.355	-	-	-	-	-	-	0.02	-	0.10	-	Disk	Thin dense white cords	-	-
G 7	2	0.355	0.01	-	-	0.01	-	-	-	-	-	-	Reisol	Fine white fluffy mycelium	-	-
G 9	2	0.355	0.01	0.01	-	-	-	-	-	-	-	-	Disk	Fine yellow cords	-	-
Hypocrea pachybasioides	2	0.355	-	0.01	-	0.01	-	-	-	-	-	-	Reisol	White mycelium, fan-like growth	FJ860789	Ascomycota
Hypoxylon serpens	2	0.355	-	-	0.02	0.01	-	-	-	-	-	-	Reisol	Green/brown pigmented mycelium	JN979420	Ascomycota
Parasola/Phlebia	2	0.355	0.01	-	-	-	-	0.03	-	-	-	-	Disk	Butter yellow thick feathery cords	-	Basidiomycota
Phoma eupyrena	2	0.355	-	-	-	-	0.04	-	-	-	-	-	Reisol	Dark grey with craggy light grey aerial mycel	HQ115670	Ascomycota
Phoma sp NF H	2	0.355	0.01	-	-	-	-	-	-	0.05	-	-	Disk	-	JX160059	Ascomycota
Sarocladium strictum	2	0.355	0.01	-	-	0.01	-	-	-	-	-	-	Reisol	Grey/brown grainy mycelium	KF675771	Ascomycota
U21	2	0.355	-	-	-	0.03	-	-	-	-	-	-	Disk	Fluffy matte yellow mycelium	-	-
uncultured Bionectria|Bionectria	2	0.355	-	-	0.03	-	-	-	-	-	-	-	Reisol	Orange/brown with lots of white aerial mycel	JF449885	Ascomycota
uncultured fungus|Mycena	2	0.355	-	-	-	0.01	-	-	-	-	-	0.02	Reisol	White mycelium with spiral margins	DQ309097	Basidiomycota
uncultured Phoma	2	0.355	-	0.01	0.02	-	-	-	-	-	-	-	Reisol	Grey with fluffy white aerial mycelium	EU852354	Ascomycota
Agaricomycetes	1	0.177	-	-	-	-	-	-	0.02	-	-	-	Cords	Fluffy white mycelium	UDB018605	Basidiomycota
Arthrinium arundinis	1	0.177	-	0.01	-	-	-	-	-	-	-	-	Reisol	Cream fluffy mycelium	KF144889	Ascomycota
Ascocoryne sarcoides	1	0.177	0.01	-	-	-	-	-	-	-	-	-	Reisol	Mulberry mycelium with halo of small colonies	HM152550	Ascomycota
Cristinia helvetica	1	0.177	-	0.01	-	-	-	-	-	-	-	-	Reisol	-	UDB016420	Basidiomycota
fungal endophyte	1	0.177	-	0.01	-	-	-	-	-	-	-	-	Reisol	Cream fluffy with bright orange aerial mycelium	KF435168	-

Taxon name/code	Occurrence		Occurrence as proportion of total cOTUs following each precoloniser species										Recovery method	Description	Accession number	Phylum
	No.	% total	Control	*V. comedens*	*H. fragiforme*	*B. nummularia*	*T. versicolor*	*S. hirsutum*	*B. adusta*	*H. fasciculare*	*P. velutina*	*P. impudicus*				
fungal sp F12|Mortierella	1	0.177	-	-	-	-	-	-	-	-	0.10	-	Reisol	Dense white with orange/brown centre	EU725674	Ascomycota
fungal sp NLEndoHerit 016 2008N1 28 3E	1	0.177	-	0.01	-	-	-	-	-	-	-	-	Reisol	White flat mycelium with concentric zones of clearing around the inoculum	JX978245	-
Gibberella zeae	1	0.177	-	-	-	0.01	-	-	-	-	-	-	Reisol	Bright pink/yellow fluffy mycelium	-	-
Gymnopus peronatus	1	0.177	-	-	0.02	-	-	-	-	-	-	-	Disk	-	HQ333195	Ascomycota
Hypocrea koningii	1	0.177	-	-	-	0.01	-	-	-	-	-	-	Reisol	Fluffy white mycelium	UDB011029	Ascomycota
Hypocrea lixii	1	0.177	-	-	-	-	-	-	0.02	-	-	-	Disk	Fluffy yellow mycelium with clumped cords at edges	X93984	Ascomycota
Hypocrea neorufoides	1	0.177	-	-	-	-	-	-	0.02	-	-	-	Reisol	Yellow/cream fluffy mycelium, pigmented bro	U78881	Ascomycota
Hypocrea viridescens	1	0.177	0.01	-	-	-	-	-	-	-	-	-	Reisol	Cream fluffy mycelium	GU062213	Ascomycota
Hypoxylon fragiforme	1	0.177	-	-	-	-	-	-	-	0.05	-	-	Reisol	Dark green/brown hard balls	GU566274	Ascomycota
Ilyonectria radicicola	1	0.177	-	-	0.02	-	-	-	-	-	-	-	Reisol	Fine white mycelium with green/brown spore:	HM036598	Ascomycota
Lewia infectoria	1	0.177	-	-	-	0.01	-	-	-	-	-	-	Disk	-	KC292865	Ascomycota
Mortierella sp WD2G	1	0.177	-	-	-	-	-	-	-	0.05	-	-			JX270447	Ascomycota
Mucor genevensis	1	0.177	-	-	0.02	-	-	-	-	-	-	-	Reisol	Fine white mycelium with green/brown spore:	EU484296	Ascomycota
Mycena citrinomarginata	1	0.177	-	-	-	-	-	-	-	-	-	0.02	Disk	-	JF908416	Basidiomycota
Mycena sp MCVE 997	1	0.177	-	-	-	-	0.02	-	-	-	-	-	Disk	Yellow/white mycelium	JF908495	Basidiomycota
Nectria nigrescens	1	0.177	-	0.01	-	-	-	-	-	-	-	-	Reisol	-	HM484550	Ascomycota
Nemania serpens	1	0.177	-	-	-	0.01	-	-	-	-	-	-	Reisol	-	HQ115648	Ascomycota
Neonectria sp.	1	0.177	-	-	-	-	-	-	-	-	-	0.02	Reisol	Patchy white mycelium with grey spores in older regions	KC758704	Ascomycota
Phialocephala dimorphospora	1	0.177	-	-	-	-	0.02	-	-	-	-	-	Reisol	Bright yellow flat mycelium	AY606309	Ascomycota
Rhinocladiella similis	1	0.177	-	-	0.02	-	-	-	-	-	-	-	Reisol	-	KC254071	Ascomycota
soil fungal sp ANG28	1	0.177	-	-	-	0.01	-	-	-	-	-	-	Reisol	Grey fluffy mycelium	DQ914420	-
Trichoderma viridarium	1	0.177	-	0.01	-	-	-	-	-	-	-	-	Reisol	Green bobbly mycelium with lots of spores	X93987	Ascomycota
U1	1	0.177	0.01	-	-	-	-	-	-	-	-	-	Disk	Bright orange mycelium	-	-
U10	1	0.177	-	-	0.02	-	-	-	-	-	-	-	Disk	Cream mycelium	-	-
U11	1	0.177	-	0.01	-	-	-	-	-	-	-	-	Disk	Cream spiny cords	-	-
U12	1	0.177	0.01	-	-	-	-	-	-	-	-	-	Disk	Cream/brown balls	-	-
U13	1	0.177	-	-	-	-	-	-	-	-	-	0.02	Disk	Dark orange mycelium with yellow cords	-	-
U14	1	0.177	0.01	-	-	-	-	-	-	-	-	-	Disk	Fine white aerial mycelium	-	-
U15	1	0.177	-	-	-	-	0.02	-	-	-	-	-	Reisol	Fine, feathery white mycelium	-	-
U16	1	0.177	0.01	-	-	-	-	-	-	-	-	-	Reisol	Flat cream mycelium with fine cords	-	-
U17	1	0.177	-	-	-	0.01	-	-	-	-	-	-	Reisol	Flat white mycelium with orange pigmentatio	-	-
U18	1	0.177	-	-	-	0.01	-	-	-	-	-	-	Reisol	Flat white mycelium, slighty pigmented	-	-
U19	1	0.177	-	-	-	-	-	-	-	-	-	0.02	Reisol	Fluffy brown mycelium	-	-
U2	1	0.177	0.01	-	-	-	-	-	-	-	-	-	Disk	Black bobbly mycelium	-	-
U20	1	0.177	-	-	-	-	-	-	0.02	-	-	-	Disk	Fluffy grey mycelium	-	-
U22	1	0.177	0.01	-	-	-	-	-	-	-	-	-	Disk	Fluffy white mycelium with orange spines	-	-
U23	1	0.177	0.01	-	-	-	-	-	-	-	-	-	Disk	Fluffy yello/white mycellium	-	-
U24	1	0.177	-	-	-	0.01	-	-	-	-	-	-	Reisol	Green/black with lots of aerial mycelium	-	-
U25	1	0.177	-	-	0.02	-	-	-	-	-	-	-	Reisol	Grey fluffy mycelium with lots of spores	-	-
U26	1	0.177	-	0.01	-	-	-	-	-	-	-	-	Disk	Light grey spores	-	-
U27	1	0.177	-	-	-	-	-	-	-	0.05	-	-	Disk	Light orange mycelium with spores	-	-
U28	1	0.177	-	-	-	-	0.02	-	-	-	-	-	Disk	Matte orange mycelium with yellow cords	-	-
U3	1	0.177	-	-	-	-	-	-	-	-	-	0.02	Disk	Black velvety mycelium	-	-
U30	1	0.177	-	-	-	-	-	-	-	-	-	0.02	Disk	Orange mycelium with spores	-	-
U31	1	0.177	0.01	-	-	-	-	-	-	-	-	-	Reisol	Orange mycelium with white fluffy edges	-	-
U32	1	0.177	0.01	-	-	-	-	-	-	-	-	-	Disk	Orange/yellow fluffy mycelium	-	-
U33	1	0.177	-	-	-	-	0.02	-	-	-	-	-	Disk	Orange/yellow rubbery flat mycelium	-	-
U34	1	0.177	-	-	0.02	-	-	-	-	-	-	-	Disk	Orange/yellow thick cords	-	-

Taxon name/code	Occurrence		Occurrence as proportion of total cOTUs following each precoloniser species										Recovery method	Description	Accession number	Phylum
	**No.**	**% total**	**Control**	***V. comedens***	***H. fragiforme***	***B. nummularia***	***T. versicolor***	***S. hirsutum***	***B. adusta***	***H. fasciculare***	***P. velutina***	***P. impudicus***				
U35	1	0.177	-	-	-	-	0.02	-	-	-	-	-	Disk	Pale pink and green mycelium with spores	-	-
U36	1	0.177	0.01	-	-	-	-	-	-	-	-	-	Disk	Pale yellow fluffy mycelium	-	-
U37	1	0.177	-	0.01	-	-	-	-	-	-	-	-	Reisol	Penicillium-type	-	-
U38	1	0.177	-	-	-	0.01	-	-	-	-	-	-	Reisol	Pink/orange mycelium with green spores	-	-
U39	1	0.177	-	-	-	-	0.02	-	-	-	-	-	Reisol	Red mycelium	-	-
U4	1	0.177	-	-	-	0.01	-	-	-	-	-	-	Disk	Bright orange zone lines and pimentation	-	-
U40	1	0.177	-	-	0.02	-	-	-	-	-	-	-	Disk	Rust-coloured, patchy mycelium	-	-
U41	1	0.177	-	-	-	0.01	-	-	-	-	-	-	Disk	Thick cream mycelium	-	-
U42	1	0.177	0.01	-	-	-	-	-	-	-	-	-	Disk	Thick white bobbly mycelium	-	-
U43	1	0.177	0.01	-	-	-	-	-	-	-	-	-	Disk	Transparent fluffy mycelium	-	-
U44	1	0.177	-	-	-	-	0.02	-	-	-	-	-	Reisol	Transparent, flat mycelium	-	-
U45	1	0.177	-	-	-	0.01	-	-	-	-	-	-	Disk	White /green fan-like mycelium	-	-
U46	1	0.177	0.01	-	-	-	-	-	-	-	-	-	Disk	White and orange cords	-	-
U47	1	0.177	-	-	0.02	-	-	-	-	-	-	-	Disk	White cords like cobweb	-	-
U48	1	0.177	-	-	-	-	-	0.03	-	-	-	-	Disk	White feathery cords	-	-
U49	1	0.177	-	-	-	-	0.02	-	-	-	-	-	Disk	White feathery cords, orange pigmentation	-	-
U5	1	0.177	-	-	-	0.01	-	-	-	-	-	-	Reisol	Bright yellow/green fluffy mycelium	-	-
U50	1	0.177	-	0.01	-	-	-	-	-	-	-	-	Reisol	White flat mycelium	-	-
U51	1	0.177	-	-	-	-	-	-	-	-	-	0.02	Disk	White mycelium with yellow cords	-	-
U52	1	0.177	0.01	-	-	-	-	-	-	-	-	-	Disk	White/cream mycelium in column through disk	-	-
U53	1	0.177	-	-	0.02	-	-	-	-	-	-	-	Disk	Woolly cream mycelium	-	-
U54	1	0.177	-	0.01	-	-	-	-	-	-	-	-	Disk	Yellow mycelium with spores	-	-
U55	1	0.177	0.01	-	-	-	-	-	-	-	-	-	Disk	Yellow spiny cords	-	-
U56	1	0.177	-	-	-	-	0.02	-	-	-	-	-	Reisol	Yellow thick mycelium	-	-
U57	1	0.177	-	-	-	-	-	-	0.02	-	-	-	Reisol	Yellow-edged, brown centred cords	-	-
U58	1	0.177	-	-	0.02	-	-	-	-	-	-	-	Reisol	Yellow/cream mycelium	-	-
U59	1	0.177	-	-	-	-	-	-	-	0.05	-	-	Disk	Yellow/orange cords with lighter yellow edge	-	-
U6	1	0.177	-	0.01	-	-	-	-	-	-	-	-	Disk	Cream and orange cords	-	-
U60	1	0.177	-	-	-	0.01	-	-	-	-	-	-	Disk	Yellow/orange haphazard cords	-	-
U61	1	0.177	-	-	0.02	-	-	-	-	-	-	-	Disk	Yellow/orange mycelium in column through d	-	-
U7	1	0.177	0.01	-	-	-	-	-	-	-	-	-	Reisol	Cream bobbly mycelium	-	-
U8	1	0.177	-	-	-	0.01	-	-	-	-	-	-	Disk	Cream cords with yellow edges	-	-
U9	1	0.177	0.01	-	-	-	-	-	-	-	-	-	Disk	Cream cords, highly branched	-	-
uncultured Agaricaceae	1	0.177	-	-	0.02	-	-	-	-	-	-	-	Disk	-	AM076650	Basidiomycota
uncultured Arthrinium	1	0.177	-	-	-	-	-	-	0.02	-	-	-	Reisol	White aerial mycelium with antler-like protuberances	KF385288	Ascomycota
uncultured endophytic fungus	1	0.177	-	-	-	-	-	-	0.02	-	-	-	Cords	Brigh orange fluffy mycelium, highly pigmented	EF505343	-
uncultured fungus|Mortierella	1	0.177	-	-	-	0.01	-	-	-	-	-	-	Reisol	-	JX316324	Ascomycota
uncultured fungus|Umbelopsis	1	0.177	-	-	-	0.01	-	-	-	-	-	-	Reisol	-	EF434088	Ascomycota
uncultured Melanopsammella	1	0.177	-	-	-	-	-	-	0.02	-	-	-	Disk	-	JF519556	Ascomycota
uncultured Mucorales|Umbelopsis	1	0.177	-	-	0.02	-	-	-	-	-	-	-	Reisol	Flat dense white mycelium, puckered in centre	JF691265	Ascomycota
uncultured Trichoderma	1	0.177	-	0.01	-	-	-	-	-	-	-	-	Reisol	-	JF519023	Ascomycota
		Number of OTUs	51	46	42	48	36	21	34	16	10	32				
		Number of counts	119	98	65	68	57	29	52	20	10	46				
