# Supplementary material for: Priority effects during fungal community establishment in beech wood
Source: ISME J. 2015 Mar 20;9(10):2246–60. doi: 10.1038/ismej.2015.38 (PMC4579477; doi:10.1038/ismej.2015.38)
Supplement: Supplementary Table 6 [file ismej201538x10.pdf]

Supplementary Table 6. OTUs responsible for the top 50% variability between precoloniser species. Pairwise comparisons were performed using Adonis/PERMANOVA, following which a P-adjustment was performed to reduce the effect of false discovery rates (Benjamini and Holden 1995); adjusted P-values are shown. '*' indicates a significant difference in community composition (P<0.05). Adonis-Simper tests were conducted to determine which OTUs were responsible for 50% of the variability between pairwise community profiles.

**Pairwise comparison**	**Adonis outcomes**			**Adonissimper results OTU ID**									
	**df**	**F**	**P (adj)**	**No. OTUs**	**Contrast**	**SD**	**Ratio**	**Av. % A OTUs**	**Av. % B OTUs**	**Cumulative variation**	**OTU ID**	**Identification**	**Taxonomy**
Control – *V. comedens*	16	2.510	0.014 *	6	0.15	0.19	0.78	54.75	2.11	0.15	scata_0	*Lasiosphaeris hispida*	Ascomycota
					0.09	0.17	0.51	18.38	19.89	0.25	scata_9	*Coprinellus impatiens*	Basidiomycota
					0.08	0.14	0.58	0.50	32.44	0.33	scata_3	*Helotiales* sp SGSGf25	Ascomycota
					0.07	0.12	0.64	9.13	22.78	0.41	scata_4	*Xenasmatella vaga*	Basidiomycota
					0.05	0.04	1.23	20.25	0.78	0.46	Scata_2	uncultured *Ascomycete*	Ascomycota
					0.05	0.08	0.65	0.13	19.67	0.52	scata_17	*Hypocrea pachybasioides*	Ascomycota
Control – *H. fragiforme*	16	0.840	0.654	6	0.16	0.17	0.97	54.75	36.22	0.19	scata_0	*Lasiosphaeris hispida*	Ascomycota
					0.08	0.09	0.80	20.25	26.00	0.28	scata_2	uncultured *Ascomycete*	Ascomycota
					0.06	0.09	0.65	9.13	20.00	0.35	scata_4	*Xenasmatella vaga*	Basidiomycota
					0.06	0.12	0.49	18.38	8.11	0.42	scata_9	*Coprinellus impatiens*	Basidiomycota
					0.05	0.09	0.55	0.25	19.44	0.48	scata_7	*Chalara* sp mh10666	Ascomycota
					0.04	0.12	0.35	0.00	17.11	0.53	scata_58	uncultured *Agaricaceae*	Basidiomycota
Control – *B. nummularia*	15	0.749	0.750	5	0.16	0.16	1.00	54.75	43.38	0.19	scata_0	*Lasiosphaeris hispida*	Ascomycota
					0.09	0.18	0.53	18.38	25.13	0.30	scata_9	*Coprinellus impatiens*	Basidiomycota
					0.08	0.07	1.13	20.25	31.38	0.40	scata_2	uncultured *Ascomycete*	Ascomycota
					0.06	0.17	0.39	0.13	26.38	0.47	scata_15	*Coprinopsis lagopus*	Basidiomycota
					0.06	0.16	0.38	0.00	24.75	0.54	scata_48	*Coprinopsis marcescibilis*	Basidiomycota
Control – *T. versicolor*	15	2.786	0.011 *	6	0.17	0.22	0.77	54.75	3.00	0.18	scata_0	*Lasiosphaeris hispida*	Ascomycota
					0.08	0.09	0.84	2.25	29.00	0.26	scata_1	*Phialocephala dimorphospora*	Ascomycota
					0.07	0.14	0.54	9.13	18.13	0.34	scata_4	*Xenasmatella vaga*	Basidiomycota
					0.07	0.05	1.27	20.25	0.00	0.41	scata_2	uncultured *Ascomycete*	Ascomycota
					0.06	0.16	0.37	18.38	0.00	0.47	scata_9	*Coprinellus impatiens*	Basidiomycota
					0.05	0.12	0.43	15.88	0.00	0.53	scata_34	*Mycena galopus*	Basidiomycota
Control – *S. hirsutum*	15	3.545	0.011 *	6	0.19	0.24	0.78	54.75	0.75	0.20	scata_0	*Lasiosphaeris hispida*	Ascomycota
					0.09	0.18	0.50	18.38	11.00	0.29	scata_9	*Coprinellus impatiens*	Basidiomycota
					0.07	0.07	1.09	2.25	24.00	0.37	scata_1	*Phialocephala dimorphospora*	Ascomycota
					0.07	0.06	1.25	20.25	0.88	0.44	scata_2	uncultured *Ascomycete*	Ascomycota
					0.06	0.13	0.43	15.88	0.00	0.49	scata_34	*Mycena galopus*	Basidiomycota
					0.04	0.12	0.37	12.38	0.13	0.54	scata_41	*Parasola conopilus*	Basidiomycota
Control – *B. adusta*	16	2.839	0.012 *	6	0.17	0.20	0.85	54.75	21.33	0.19	scata_0	*Lasiosphaeris hispida*	Ascomycota
					0.08	0.12	0.72	9.13	28.11	0.28	scata_4	*Xenasmatella vaga*	Basidiomycota
					0.08	0.08	0.99	0.63	26.89	0.37	scata_8	*Chaetosphaeria innumera*	Ascomycota
					0.06	0.05	1.24	20.25	1.00	0.43	scata_2	uncultured *Ascomycete*	Ascomycota
					0.05	0.15	0.37	18.38	0.11	0.49	scata_9	*Coprinellus impatiens*	Basidiomycota
					0.05	0.11	0.43	15.88	0.00	0.54	scata_34	*Mycena galopus*	Basidiomycota
Control – *H. fasciculare*	11	2.193	0.026 *	5	0.18	0.25	0.74	54.75	1.50	0.19	scata_0	*Lasiosphaeris hispida*	Ascomycota
					0.15	0.16	0.95	9.13	59.50	0.35	scata_4	*Xenasmatella vaga*	Basidiomycota
					0.07	0.06	0.17	20.25	0.50	0.43	scata_2	uncultured *Ascomycete*	Ascomycota
					0.06	0.18	0.36	18.38	0.00	0.49	scata_9	*Coprinellus impatiens*	Basidiomycota
					0.05	0.13	0.41	15.88	0.00	0.55	scata_34	*Mycena galopus*	Basidiomycota
V. comedens – H. fragiforme	17	1.844	0.031 *	6	0.10	0.17	0.61	2.11	36.22	0.11	scata_0	*Lasiosphaeris hispida*	Ascomycota
					0.10	0.13	0.75	22.78	20.00	0.21	scata_4	*Xenasmatella vaga*	Basidiomycota
					0.08	0.14	0.57	32.44	0.11	0.30	scata_3	*Helotiales* sp SGSGf25	Ascomycota
					0.07	0.13	0.55	0.78	26.00	0.37	scata_2	uncultured *Ascomycete*	Ascomycota
					0.07	0.15	0.47	19.89	8.11	0.45	scata_9	*Coprinellus impatiens*	Basidiomycota
					0.06	0.10	0.62	6.11	19.44	0.51	scata_7	*Chalara* sp mh10666	Ascomycota
V. comedens – B. nummularia	16	2.058	0.018 *	6	0.12	0.18	0.65	2.11	43.38	0.12	scata_0	*Lasiosphaeris hispida*	Ascomycota
					0.10	0.21	0.50	19.89	25.13	0.23	scata_9	*Coprinellus impatiens*	Basidiomycota
					0.08	0.11	0.74	0.78	31.38	0.32	scata_2	uncultured *Ascomycete*	Ascomycota
					0.08	0.14	0.57	32.44	0.00	0.40	scata_3	*Helotiales* sp SGSGf25	Ascomycota
					0.07	0.19	0.38	0.00	26.38	0.47	scata_15	*Coprinopsis lagopus*	Basidiomycota
					0.07	0.18	0.37	0.00	24.75	0.54	scata_48	*Coprinopsis marcescibilis*	Basidiomycota
V. comedens – T. versicolor	16	1.818	0.022 *	5	0.13	0.19	0.66	22.78	18.13	0.14	scata_4	*Xenasmatella vaga*	Basidiomycota
					0.11	0.19	0.56	32.44	0.00	0.25	scata_3	*Helotiales* sp SGSGf25	Ascomycota

Pairwise comparison	Adonis outcomes			Adonissimper results OTU ID									
	df	F	P (adj)	No. OTUs	Contrast	SD	Ratio	Av. % A OTUs	Av. % B OTUs	Cumulative variation	OTU ID	Identification	Taxonomy
					0.09	0.11	0.86	5.11	29.00	0.35	scata_1	*Phialocephala dimorphospora*	Ascomycota
					0.08	0.12	0.63	19.67	1.38	0.44	scata_17	*Hypocrea pachybasioides*	Ascomycota
					0.06	0.19	0.34	19.89	0.00	0.51	scata_9	*Coprinellus impatiens*	Basidiomycota
V. comedens – S. hirsutum	16	2.377	0.011 *	5	0.11	0.20	0.56	32.44	0.13	0.12	scata_3	*Helotiales* sp SGSGf25	Ascomycota
					0.10	0.18	0.55	22.78	2.88	0.23	scata_4	*Xenasmatella vaga*	Basidiomycota
					0.10	0.21	0.48	19.89	11.00	0.34	scata_9	*Coprinellus impatiens*	Basidiomycota
					0.09	0.09	0.99	5.11	24.00	0.43	scata_1	*Phialocephala dimorphospora*	Ascomycota
					0.08	0.14	0.61	19.67	0.38	0.52	scata_17	*Hypocrea pachybasioides*	Ascomycota
V. comedens – B. adusta	17	1.874	0.031 *	5	0.13	0.16	0.78	22.78	28.11	0.14	scata_4	*Xenasmatella vaga*	Basidiomycota
					0.10	0.17	0.56	32.44	0.78	0.25	scata_3	*Helotiales* sp SGSGf25	Ascomycota
					0.10	0.11	0.89	0.67	26.89	0.35	scata_8	*Chaetosphaeria innumera*	Ascomycota
					0.07	0.10	0.69	19.67	3.33	0.43	scata_17	*Hypocrea pachybasioides*	Ascomycota
					0.06	0.15	0.43	18.11	4.44	0.50	scata_13	*Phanerochaete sp.*	Basidiomycota
V. comedens – H. fasciculare	12	1.412	0.144	4	0.21	0.21	0.00	22.78	59.50	0.23	scata_4	*Xenasmatella vaga*	Basidiomycota
					0.11	0.21	0.54	32.44	0.00	0.35	scata_3	*Helotiales* sp SGSGf25	Ascomycota
					0.08	0.14	0.57	19.67	0.75	0.44	scata_17	*Hypocrea pachybasioides*	Ascomycota
					0.07	0.21	0.33	19.89	0.00	0.51	scata_9	*Coprinellus impatiens*	Basidiomycota
H.fragiforme – B. nummularia	16	0.661	0.873	5	0.15	0.17	0.88	36.22	43.38	0.17	scata_0	*Lasiosphaeris hispida*	Ascomycota
					0.11	0.11	0.93	26.00	31.38	0.29	scata_2	uncultured *Ascomycete*	Ascomycota
					0.08	0.17	0.47	8.11	25.13	0.38	scata_9	*Coprinellus impatiens*	Basidiomycota
					0.07	0.17	0.43	2.78	26.38	0.46	scata_15	*Coprinopsis lagopus*	Basidiomycota
					0.06	0.17	0.38	0.11	24.75	0.53	scata_48	*Coprinopsis marcescibilis*	Basidiomycota
H. fragiforme – T. versicolor	16	1.961	0.019 *	5	0.12	0.20	0.62	36.22	3.00	0.13	scata_0	*Lasiosphaeris hispida*	Ascomycota
					0.10	0.16	0.65	20.00	18.13	0.24	scata_4	*Xenasmatella vaga*	Basidiomycota
					0.09	0.09	0.97	9.67	29.00	0.34	scata_1	*Phialocephala dimorphospora*	Ascomycota
					0.09	0.16	0.56	26.00	0.00	0.43	scata_2	uncultured *Ascomycete*	Ascomycota
					0.07	0.12	0.54	19.44	0.63	0.50	scata_7	*Chalara sp mh10666*	Ascomycota
H. fragiforme – S. hirsutum	16	2.600	0.011 *	6	0.13	0.22	0.61	36.22	0.75	0.14	scata_0	*Lasiosphaeris hispida*	Ascomycota
					0.09	0.17	0.55	26.00	0.88	0.24	scata_2	uncultured *Ascomycete*	Ascomycota
					0.08	0.07	0.15	9.67	24.00	0.33	scata_1	*Phialocephala dimorphospora*	Ascomycota
					0.07	0.13	0.57	20.00	2.88	0.40	scata_4	*Xenasmatella vaga*	Basidiomycota
					0.07	0.13	0.54	19.44	0.13	0.48	scata_7	*Chalara sp mh10666*	Ascomycota
					0.06	0.18	0.34	17.11	0.00	0.55	scata_58	*uncultured Agaricaceae*	Basidiomycota
H. fragiforme – B. adusta	17	2.029	0.052	5	0.14	0.19	0.73	36.22	21.33	0.15	scata_0	*Lasiosphaeris hispida*	Ascomycota
					0.11	0.13	0.79	20.00	28.11	0.27	scata_4	*Xenasmatella vaga*	Basidiomycota
					0.08	0.09	0.98	0.67	26.89	0.36	scata_8	*Chaetosphaeria innumera*	Ascomycota
					0.08	0.14	0.55	26.00	1.00	0.45	scata_2	uncultured *Ascomycete*	Ascomycota
					0.07	0.11	0.61	19.44	5.44	0.53	scata_7	*Chalara sp mh10666*	Ascomycota
H. fragiforme – H. fasciculare	12	1.553	0.120	4	0.17	0.18	0.99	20.00	59.50	0.19	scata_4	*Xenasmatella vaga*	Basidiomycota
					0.13	0.22	0.58	36.22	1.50	0.33	scata_0	*Lasiosphaeris hispida*	Ascomycota
					0.09	0.17	0.53	26.00	0.50	0.43	scata_2	uncultured *Ascomycete*	Ascomycota
					0.07	0.13	0.52	19.44	1.25	0.50	scata_7	*Chalara sp mh10666*	Ascomycota
B. nummularia – T. versicolor	15	2.148	0.011 *	5	0.14	0.21	0.66	43.38	3.00	0.15	scata_0	*Lasiosphaeris hispida*	Ascomycota
					0.10	0.14	0.74	31.38	0.00	0.25	scata_2	uncultured *Ascomycete*	Ascomycota
					0.09	0.23	0.38	26.38	0.00	0.34	scata_15	*Coprinopsis lagopus*	Basidiomycota
					0.08	0.09	0.92	6.50	29.00	0.43	scata_1	*Phialocephala dimorphospora*	Ascomycota
					0.08	0.22	0.37	25.13	0.00	0.51	scata_9	*Coprinellus impatiens*	Basidiomycota
B. nummularia – S. hirsutum	15	2.675	0.012 *	5	0.15	0.23	0.65	43.38	0.75	0.16	scata_0	*Lasiosphaeris hispida*	Ascomycota
					0.11	0.24	0.48	25.13	11.00	0.27	scata_9	*Coprinellus impatiens*	Basidiomycota
					0.11	0.15	0.75	31.38	0.88	0.39	scata_2	uncultured *Ascomycete*	Ascomycota
					0.09	0.24	0.38	26.38	0.00	0.48	scata_15	*Coprinopsis lagopus*	Basidiomycota
					0.09	0.23	0.37	24.75	0.13	0.57	scata_48	*Coprinopsis marcescibilis*	Basidiomycota
B. nummularia – B. adusta	16	2.432	0.016 *	5	0.15	0.20	0.76	43.38	21.33	0.16	scata_0	*Lasiosphaeris hispida*	Ascomycota
					0.09	0.13	0.74	31.38	1.00	0.26	scata_2	uncultured *Ascomycete*	Ascomycota
					0.08	0.08	1.00	0.00	26.89	0.34	scata_8	*Chaetosphaeria innumera*	Ascomycota
					0.08	0.21	0.38	26.38	0.00	0.43	scata_15	*Coprinopsis lagopus*	Basidiomycota
					0.08	0.20	0.37	25.13	0.11	0.50	scata_9	*Coprinellus impatiens*	Basidiomycota

Pairwise comparison	Adonis outcomes			Adonissimper results OTU ID									
	df	F	P (adj)	No. OTUs	Contrast	SD	Ratio	Av. % A OTUs	Av. % B OTUs	Cumulative variation	OTU ID	Identification	Taxonomy
B. nummularia – H. fasciculare	11	1.875	0.017 *	5	0.15	0.24	0.63	43.38	1.50	0.15	scata_0	*Lasiosphaeris hispida*	Ascomycota
					0.14	0.16	0.88	0.13	59.50	0.30	scata_4	*Xenasmatella vaga*	Basidiomycota
					0.11	0.15	0.71	31.38	0.50	0.41	scata_2	uncultured *Ascomycete*	Ascomycota
					0.09	0.25	0.37	26.38	0.00	0.50	scata_15	*Coprinopsis lagopus*	Basidiomycota
					0.09	0.24	0.36	25.13	0.00	0.59	scata_9	*Coprinellus impatiens*	Basidiomycota
T. versicolor – S. hirsutum	15	1.439	0.017 *	6	0.16	0.14	0.15	29.00	24.00	0.19	scata_1	*Phialocephala dimorphospora*	Ascomycota
					0.09	0.20	0.44	18.13	2.88	0.29	scata_4	*Xenasmatella vaga*	Basidiomycota
					0.06	0.08	0.78	0.13	14.00	0.36	scata_19	*Hyphodiscus hymeniophilus*	Ascomycota
					0.05	0.13	0.37	0.00	11.00	0.42	scata_9	*Coprinellus impatiens*	Basidiomycota
					0.05	0.05	0.88	8.00	5.13	0.47	scata_8	*Chaetosphaeria innumera*	Ascomycota
					0.03	0.07	0.52	5.38	0.50	0.51	scata_51	*Chaetosphaeria chloroconia*	Ascomycota
T. versicolor – B. adusta	16	1.439	0.144	4	0.14	0.20	0.68	18.13	28.11	0.16	scata_4	*Xenasmatella vaga*	Basidiomycota
					0.12	0.13	0.93	29.00	15.67	0.31	scata_1	*Phialocephala dimorphospora*	Ascomycota
					0.12	0.13	0.94	8.00	26.89	0.45	scata_8	*Chaetosphaeria innumera*	Ascomycota
					0.08	0.15	0.50	3.00	21.33	0.54	scata_0	*Lasiosphaeris hispida*	Ascomycota
T. versicolor – H. fasciculare	11	1.137	0.356	4	0.23	0.26	0.89	18.13	59.50	0.27	scata_4	*Xenasmatella vaga*	Basidiomycota
					0.13	0.15	0.87	29.00	8.75	0.42	scata_1	*Phialocephala dimorphospora*	Ascomycota
					0.05	0.06	0.88	8.00	5.75	0.47	scata_8	*Chaetosphaeria innumera*	Ascomycota
					0.04	0.05	0.82	0.13	6.75	0.51	scata_68	*Helotiales sp JP2013*	Ascomycota
S. hirsutum – B. adusta	16	2.175	0.019 *	5	0.13	0.15	0.90	5.13	26.89	0.15	scata_8	*Chaetosphaeria innumera*	Ascomycota
					0.12	0.11	0.05	24.00	15.67	0.29	scata_1	*Phialocephala dimorphospora*	Ascomycota
					0.10	0.16	0.64	2.88	28.11	0.41	scata_4	*Xenasmatella vaga*	Basidiomycota
					0.08	0.17	0.45	0.75	21.33	0.49	scata_0	*Lasiosphaeris hispida*	Ascomycota
					0.06	0.07	0.78	14.00	0.33	0.56	scata_19	*Hyphodiscus hymeniophilus*	Ascomycota
S. hirsutum – H. fasciculare	11	1.933	0.026 *	4	0.22	0.23	0.92	2.88	59.50	0.24	scata_4	*Xenasmatella vaga*	Basidiomycota
					0.13	0.14	0.94	24.00	8.75	0.38	scata_1	*Phialocephala dimorphospora*	Ascomycota
					0.07	0.09	0.76	14.00	0.00	0.46	scata_19	*Hyphodiscus hymeniophilus*	Ascomycota
					0.05	0.15	0.35	11.00	0.00	0.51	scata_9	*Coprinellus impatiens*	Basidiomycota
B. adusta – H. fasciculare	12	1.236	0.306	4	0.22	0.21	0.00	28.11	59.50	0.25	scata_4	*Xenasmatella vaga*	Basidiomycota
					0.13	0.15	0.86	26.89	5.75	0.41	scata_8	*Chaetosphaeria innumera*	Ascomycota
					0.08	0.11	0.72	15.67	8.75	0.50	scata_1	*Phialocephala dimorphospora*	Ascomycota
					0.08	0.17	0.46	21.33	1.50	0.59	scata_0	*Lasiosphaeris hispida*	Ascomycota
